# Perfectionism and Emotional Intelligence: A Person-Centered Approach

**DOI:** 10.1155/2022/8660575

**Published:** 2022-10-29

**Authors:** María Vicent, Ricardo Sanmartín, Nancy Isabel Cargua-García, José Manuel García-Fernández

**Affiliations:** ^1^Department of Developmental Psychology and Teaching, Faculty of Education, University of Alicante, Apdo. Correos 99 E-03080, Alicante, Spain; ^2^Department of Developmental Psychology and Teaching, University of Alicante, Carretera San Vicente Del Raspeig S/n 03690 San Vicente Del Raspeig, Alicante, Spain; ^3^Faculty of Philosophy, Literature and Educational Sciences, Central University of Ecuador, Cuidadela Universitaria, Av. América, Quito, Ecuador

## Abstract

This study examined the relationship between perfectionistic concerns (PC) and perfectionistic strivings (PS) with the subcomponents of emotional intelligence (EI) through a latent class person-centered approach. A sample of 1582 Ecuadorian adolescents (619 females) aged from 12 to 18 was employed. The trait meta-mood scale-24 (TMMS-24) and the child and adolescent perfectionism scale (CAPS) were used, respectively, for assessing three subcomponents of EI (i.e., emotional attention, emotional clarity, and mood repair) and two perfectionist dimensions (PC and PS). A three-class solution (High perfectionism, moderate perfectionism, and nonperfectionism) was identified by using latent class analysis. High perfectionism significantly scored higher on emotional attention in comparison with the moderate and nonperfectionism classes, with small and moderate effect sizes. Overall, results suggest that people with high perfectionism might be at greater risk of developing maladaptive emotional self-regulation strategies, such as rumination, because of their tendency to excessively attend their negative mood states.

## 1. Introduction

Perfectionism is a multidimensional trait of personality characterized by the pursuit of perfection and the establishment of extremely high-performance standards, accompanied by overly critical evaluations of oneself and others, as well as beliefs about perfectionist demands and criticisms of the significant others [1–3]. This personality trait “matters a great deal” not only because it has been associated with a large number of psychological problems [4], but also because of its considerable increasing prevalence in society [5, 6]. Although perfectionism has been mainly considered as a negative construct due to its close relationship with overall psychopathology [7], several authors defend the existence of both positive and negative perfectionist dimensions [8]. Thus, the two-factor model proposes that perfectionist dimensions can be organized into two higher-order factors commonly labelled as perfectionistic strivings (PS) and perfectionistic concerns (PC). PS represents the adaptive, or at least not maladaptive, dimension of perfectionism, and it reflects the need to be perfect and to follow excessively high goals, whereas PC involves constant and harsh self-criticism, chronic concerns about others' criticisms and expectations, and the impossibility of experiencing satisfaction from successful performances, representing the maladaptive dimension of perfectionism [8]. This two-factor model acts as a conceptual framework that allows comparing the results from different studies regardless of the multidimensional perfectionism scale used [2].

Emotional intelligence (EI) refers to abilities that “enable awareness of the emotional states of oneself and others and the capacity to regulate or use emotions to positively affect role performance” (p. 140) [9]. Two predominant conceptualizations of EI exist: ability emotional intelligence (AEI) and trait emotional intelligence (TEI). AEI refers to the capacity to understand, perceive, use, and regulate emotions, which is assessed through maximum performance tests [10]. In contrast, TEI is defined by the individual's self-perceptions and dispositions as measured using self-reported scales [11].

Since it was first presented by Salovey and Mayer [12] and popularized by Goleman [13], EI has generated a high level of public and scientific interest. Currently, there is strong evidence that high levels of EI are associated with better mental, physical, and general health in both clinical and nonclinical populations [14]. EI also plays an important role in academic and job performance [15–17]. It is considered a significant predictor of long-term career success [18], life satisfaction, and subjective happiness and well-being [19, 20], as well as a significant negative health-related risk behavior [21].

### 1.1. Emotional Intelligence and Perfectionism

The link between perfectionism and EI has been addressed by previous research following both a person-centered and a variable-centered approach. Preliminary results on this topic based on a variable-centered approach indicate that PC shows consistent negative associations with EI, whereas the relationship between PS and EI is not as clear. Specifically, Smith et al. [22] analyzed, in a sample of 223 Canadian undergraduates (*M*_*age*_ = 19.06, *SD* = 2.20), the relationship between PC and EI, finding significant and negative correlations between both constructs. Authors also found that EI partially mediated the effect of neuroticism on perfectionism. On the other hand, using 520 high school students from Iran (*M*_*age*_ = 17.25, *SD* = 2.21), Abdollahi and Abu-Talib [23] obtained that EI was significantly and positively correlated with PS, as well as negatively with PC. These same correlational patterns were found by Smith et al. [24] in a sample of 645 Chinese undergraduates (*M*_*age*_ = 20.1, *SD* = 2.31). Moreover, results also showed that EI fully mediated the link between PS and satisfaction with life and partially mediated the link between PC and depression, stress and satisfaction with life. More recently, Wilson et al. [25] and Badri et al. [26] using, respectively, a sample of 277 Canadian undergraduates (*M*_*age*_ = 18.01, *SD* = 1.46) and 259 English young adults, found that both PS and PC significantly and negatively correlated with EI. In contrast, with a sample of 120 middle school children from Slovenia, Doktorová, and Varecková [27] observed nonsignificant associations between PS, PC, and EI, except for concern about making mistakes, which was the only indicator of PC negatively and significantly associated with EI.

It is important to note that all these studies fail to address the different dimensions of EI. Instead, they focus on the global score of trait-EI, although it has been confirmed that the global score is less important and informative than its subcomponents [14]. Moreover, these studies analyze the two perfectionist dimensions in isolation, instead of considering the possible combinations of PC and PS. However, examining the combinations of perfectionist dimensions could be an important contribution towards understanding how perfectionism can be both adaptive and maladaptive [28].

Gong et al. [29] tried to overcome the mentioned limitations analyzing the relationship between perfectionism and EI based on the 2 × 2 model of dispositional perfectionism [30] in a sample of 386 undergraduates from the USA. According to the results, PS significantly and positively correlated with all EI subcomponents (i.e., emotional self-regulation, appraisal of emotions of others, appraisal of self-emotions and utilization of emotions for problem solving). In contrast, PC significantly and negatively correlated with all EI subcomponents, except for the utilization of emotions for problem solving. Regarding the 2 × 2 model, results evidenced that the subtype Pure *PS* (high PS and low PC) obtained significantly higher scores on emotional self-regulation, appraisal of emotions of others, and appraisal of self-emotions than the other three subtypes. In contrast, pure PC (high PC and low PS) obtained the lowest scores on these same EI subcomponents. The Mixed subtype (high PC and PS) scored higher than Pure PC on emotional self-regulation, appraisal of emotions of others and appraisal of self-emotions; but lower than Pure PS. In the case of the subtype contrasts for the utilization of emotions for problem solving, nonsignificant differences were obtained between pure PS and the mixed subtype.

Although results based on a variable-centered approach provide useful information regarding the nature of each perfectionist dimension in terms of EI, these insights should be analyzed with caution. Greenspon [31] affirms that even though perfectionist dimensions can be studied separately in the research lab, these facets do not appear as separate constructs in lived experience. In this sense, a person-centered approach allows us to identify profiles based on different levels of perfectionist dimensions, and these profiles are, in turn, linked to different outcomes. Therefore, in accordance with Lundh et al. [32], a person-centered approach is a better reflection of real life.

Following a person-centered approach, Chan [33] identified three profiles of perfectionism in a sample of 380 Chinese gifted students (*M*_*age*_ = 12.19, *SD* = 2.18): unhealthy perfectionists, characterized by high PS and low PC; healthy perfectionists, with high scores on both PS and PC; and nonperfectionists, with low scores on both PS and PC. Overall, healthy perfectionists obtained the highest scores on all EI subcomponents, followed by unhealthy perfectionists, whereas nonperfectionists obtained the lowest scores. However, several potential difficulties have been associated with the clustering procedure (i.e., k-means) employed by Chan [33], such as the sensitivity to outliers or the need to use interval or ratio data, between others. All these problems can be easily overcome by using latent class analysis (LCA) [34]. “The person-centered LCA approach assumes the existence of mutually exclusive and exhaustive groups of individuals that can be differentiated by values of unobserved latent variables” (p. 228) [35] that are measured by observed variables.

LCA has received increasing attention over the last years as a promising method of establishing groups of perfectionists. However, the number of classes obtained by previous research based on this approach varies from one study to another. One possible explanation for these variations may be the measure of perfectionism employed. For instance, studies in which perfectionism was assessed by using the Almost Perfect Scale-Revised (APS-R) [36] usually find a three-class model: adaptive perfectionists (high PS and low PC), maladaptive perfectionists (high or low PS and high PC) and nonperfectionists (low PS and PC) [37–39]. Vicent et al. [40] also found a three-cluster solution using the Child and Adolescent Perfectionism Scale (CAPS) [41]. However, in this case, the three classes were characterized by high perfectionism (high PS and PC), moderate perfectionism (moderate PS and PC) and nonperfectionism (low PS and PC). By contrast, Herman et al. [42] obtained a four-cluster solution using a short version of the CAPS, whereas Sironic and Reeve [43], who employed three different scales of perfectionism, found a better fit for a model of six classes.

### 1.2. This Study

Even though researchers have dedicated considerable attention toward identifying the relationship between perfectionism and EI following a variable-centered approach, there is a need for clarity in the perfectionism literature regarding the differences and similarities between profiles of perfectionism in terms of EI. This is, to our knowledge, because there is only one study [33] that has addressed this issue through a person-centered approach, and, as mentioned above, it has several limitations regarding the clustering method employed.

The purpose of this study was to analyze the relationship between perfectionism and EI through a person-centered approach. LCA was employed to identify the number of classes that represent different profiles of perfectionism. Then, interclass differences in the subcomponents of EI were examined. Based on the results of previous literature that have identified classes of perfectionism using the CAPS, it is expected to find a three-class solution made of high, moderate and nonperfectionism [40]. Secondly, in the case of finding this three-class model, the High perfectionism profile is likely to score lower on EI than moderate and nonperfectionism. That is because previous research has found evidence suggesting that individuals with higher PC may have lower EI [22–25, 29].

## 2. Method

### 2.1. Participants and Procedure

Participants were recruited using a multistage random cluster sampling from 11 high schools in Quito (Ecuador). Participants consisted of 1582 students (619 females) aging from 12 to 18 years (*M* = 14.82, *SD* = 1.86). Students' socio-economic status consisted of 23% medium-low, 60% medium, and 17% medium-high.

All procedures were performed according to the ethical standards of the 1964 Helsinki Declaration. An interview with the principals of the schools was conducted in order to explain the purposes of the study and request their collaboration. Participation of the students was voluntary and written parental consent was obtained prior to data collection. Participants completed the tests anonymously during scheduled classes under the supervision of a researcher.

### 2.2. Measures

The instruments used to measure the variables of this study, i.e., perfectionism and IE, are listed below.

#### 2.2.1. Perfectionism

PC and PS were measured using, respectively, the Socially Prescribed Perfectionism and the Self-Oriented Perfectionism subscales of the Child and Adolescent Perfectionism Scale (CAPS) [41]. Specifically, the Spanish version translated by Castro et al. [44] was employed in this study. The use of Self-Oriented Perfectionism and Socially Prescribed Perfectionism as indicators of PS and PC has been supported by previous research [2]. Socially Prescribed Perfectionism (10 items; e.g., “Other people always expect me to be perfect”) refers to the belief that there are excessively high expectations and criticisms from the environment. In contrast, self-oriented perfectionism (12 items; e.g., “I try to be perfect in everything I do”) involves the drive, and requirement to be perfect and excessively hard self-evaluations. The 22 items of the CAPS were rated on a 5-point Likert scale.

Internal consistency (Cronbach's alpha) calculated for the present study was acceptable for the CAPS subscales (see Table 1).

#### 2.2.2. Emotional Intelligence

EI was assessed by using the Trait Meta-Mood Scale-24 (TMMS-24). The TMMS-24 is a short version of the TMMS-48 [45] validated by Fernández-Berrocal et al. [46] in Spain. This instrument assesses three dimensions: (a) emotional attention or the ability to observe and be aware of one's feelings (e.g., “I pay a lot of attention to how I feel”); emotional clarity or the ability to discriminate and understand one's emotional states (e.g., “I am usually clear about my feelings”); and mood repair or the ability to properly regulate of one's emotional states (e.g., “When I become upset, I remind myself of all the pleasures in life”). The TMMS-24 was rated on a 5-point Likert scale.

Internal consistency (Cronbach's alpha) calculated for the present study was also acceptable for the TMMS-24 subscales (see Table 1).

### 2.3. Data Analysis

First, descriptive statistics (means, standard deviations, bivariate correlations, and partial correlations controlling the effect of PC and PS) and Cronbach's alphas for all of the subscales were calculated.

An LCA was conducted to classify the participants according to the CAPS subscales scores (i.e., PS and PC). Model testing began by setting a single class up to six latent profile models. The fit for the LCA models was based on the Bayesian Information Criterion (BIC), where smaller numbers indicate better fit, and Entropy, where higher scores indicate great classification accuracy [34].

Next, we conducted an analysis of variance (ANOVA) to determine whether there were differences in the mean levels of the sub-components of EI (i.e., emotional attention, emotional clarity, and mood repair) across the different latent classes. Post-hoc tests using the Bonferroni method were carried out to identify between which groups there were statistically significant differences. As recommended by Cohen [47], effect sizes were calculated, designating them as small (*d* = .20–0.49), moderate (*d* = 0.50–0.79), and large (*d* ≥ 0.80).

## 3. Results

### 3.1. Descriptive Analysis

Means, standard deviations, and correlations between scores are presented in Table 1. Positive and significant bivariate and partial correlations were obtained between PS and the three EI sub-components. Similarly, positive and significant bivariate and partial correlations were found between PC and emotional attention.

### 3.2. Latent Class Analysis

LCA fit indices for two-through six-class solutions are represented in Table 2. BIC values decreased up to the two-class model and then increased for the four-to six-class model. Then, the three- and four-class models obtained the lowest BIC value. Regarding the entropy, values were also higher for the three- and four-class models, suggesting a reasonable degree of certainty in the classification. Despite these two models showing similar values of entropy, the four-class model fit the data best. This is because the BIC is considered the best indicator of the number of classes [48]. However, regarding practicality, every solution after the three-class model contained one or more small classes. For instance, in the four-class model, a fourth class was identified that represented 2% of the students in the current sample. Moreover, in terms of interpretability, subgroups emerging beyond the three-class structure were closely aligned to the classes identified within the three-class model. Consequently, considering the statistical findings and the interpretability of the results, the three-class model was selected.

**Figure 1:**
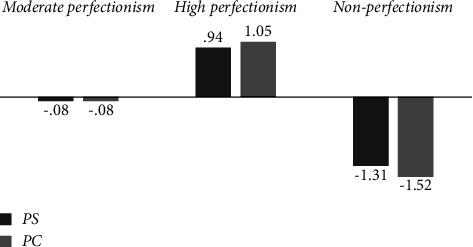
Graphic representation of the standardized average scores for the model of the latent classes.

Figure 1 shows the standardized means of PS and PC for each of the three latent classes. The first class represented 66% (*N* = 1045) of the sample and classified students with moderate scores on both PS and PC. Therefore, it was called moderate perfectionism. The second group, labelled as high perfectionism, classified the 24% (*N* = 382) of the sample characterized by high levels of PS and PC. Lastly, individuals in the third group were characterized as having low levels of PS and PC. This last group, labelled as nonperfectionism, represented 10% (*N* = 155) of the participants.

### 3.3. Inter-class Differences

Statistically significant interclass differences in terms of EI were obtained (see Table 3). The High perfectionism group showed the highest scores on all EI subcomponents. In contrast, non-perfectionism reported the lowest scores on emotional attention and emotional clarity whereas moderate perfectionism scored lowest on Mood Repair.

Post-hoc comparisons showed that high perfectionism scored significantly higher than moderate perfectionism on all EI subcomponents. Effect sizes for these differences were of a small magnitude (in the case of emotional attention) or negligible (in the case of emotional clarity and mood repair). On the other hand, high perfectionism and Moderate perfectionism scored significantly higher on emotional attention than nonperfectionism, with moderate and small effect sizes associated with these differences, respectively. Nonsignificant differences were found in the remaining interclass contrasts (see Table 4).

## 4. Discussion

This study aimed to analyze the relationship between perfectionism and EI through a latent class person-centered approach.

### 4.1. Perfectionism Profiles

Results from LCA evidenced that a three-class solution appeared to depict the most parsimonious representation of the data. This three-class solution consisted of three profiles (high perfectionism, moderate perfectionism and nonperfectionism) characterized, respectively, by high, moderate and low levels of both PS and PC. This model of three classes coincides with that found by Vicent et al. [40] in a Spanish child sample. These coincidences could be explained because both studies assessed perfectionism using a Spanish translated version of the CAPS. In fact, when our class solution is compared with that obtained by other works that assess perfectionism through the APS-R, although there are some coincidences, such as the number of classes of the nonperfectionism class (characterized by low levels of PC and PS), the two remaining classes do not match up with those obtained by the present study [37–39]. Specifically, these studies found two profiles, in addition to the nonperfectionism, commonly referred as adaptive perfectionists and maladaptive perfectionists, which were characterized by different combinations of high and low levels of PC and PS. On the contrary, in our study, a similar behavior was found for both dimensions, resulting in a high perfectionism profile and a moderate one.

In terms of prevalence, the moderate perfectionism profile classified 64% of the sample, while the high perfectionism (23%) and nonperfectionism (13%) profiles obtained a much lower prevalence. Again, these results are comparable to those obtained by Vicent et al. [40] who found that the moderate, high, and nonperfectionism classes classified 62.41%, 26.68%, and 10.90% of the sample, respectively.

### 4.2. Interprofile Differences in Terms of EI

Contrary to expectations, High perfectionism obtained the highest levels of EI, although these results were only relevant for the Emotional Attention subcomponent. Regarding the emotional clarity and mood repair subcomponents, the interclass differences were only significant for the contrast between high perfectionism and moderate perfectionism. In spite of this, the analysis of the effect sizes showed that these differences (*d* < .20), although significant, lacked practical value. By contrast, in the case of emotional attention, moderate perfectionism scored significantly lower than High perfectionism but significantly higher than nonperfectionism, with small effect sizes for both cases. Likewise, the high perfectionism group scored significantly higher than nonperfectionism, the magnitude of the differences being moderate. The effect sizes found indicated that these interclass differences in emotional attention are not only significant, but they also represent theoretical and practical relevance.

Following the advice of Stoeber [49], partial and bivariate correlations were analyzed to verify, respectively, the shared and unique relationships between the perfectionist dimensions and the subcomponents of the EI, in order to examine to what extent these interclass differences can be explained depending on the PS or PC. Bivariate correlations allow us to understand why a subject with high levels in one of the perfectionist dimensions differs or not from another individual with low levels in that perfectionist dimension. Conversely, partial correlations allow to control the superposition between PS and PC by keeping constant one of the dimensions [50, 51]. Thus, according to the results of this study, it seems that both dimensions, PS and PC, have unique and shared relationships with emotional attention, which means that both are responsible for the differences found between the three profiles.

The fact that the high perfectionism group obtained significantly higher scores than the other profiles in emotional attention, and that this was explained by the high levels of both PS and PC, is still a surprising result considering that the previous literature has provided preliminary evidence about the negative association between PC and EI [22–26, 29]. However, it must be remembered that none of these previous studies have been based on the TMMS scale. In fact, most of them used different versions of the Trait Emotional Intelligence Questionnaire Short Form (TEIQue) [11] or the Assessing Emotions Scale (AES) [52], among other scales. Although all these scales measure EI, they do not examine the same underlying dimensions and mechanisms involved [14]. In addition, most of these works focus on the analysis of EI as a general factor without taking into account the results for each of its dimensions [22–25], although it has been demonstrated that the sub-components of EI are more relevant and have much more informative value than their general factor [14].

The Emotional Attention subscale of the TMMS measures the tendency to observe and think about one's emotions and feelings, to assess their emotional states, and to maximize their emotional experiences [53]. Although certain levels of emotional attention are considered necessary to reach optimal levels of emotional regulation [54], the tendency to pay too much attention to one's emotions is not necessarily considered as an adaptive trait. In fact, high levels of emotional attention are associated with greater psychological distress, anxiety, depression, and even substance abuse [14, 55], especially when they are not accompanied by the necessary levels of clarity and emotional repair that allow the individual to understand and control their emotional states. Indeed, studies about EI profiles have shown that individuals with high levels of attention, as well as moderate and low levels of emotional clarity and mood repair, respectively, obtain worse results in terms of psychological adjustment than those profiles characterized by high levels in Mood Repair [56–58]. According to Hodzic et al. [59], stress plays a key role in the relationship between emotional attention and well-being. Apparently, the increase in stress negatively affects more students with higher levels of emotional attention than those with lower levels.

In turn, it is considered that perfectionism not only generates but also enhances stress [60]. People with high levels of perfectionism are especially vulnerable to stressors and day-to-day misfortunes. It often leads them to experience negative emotions, such as shame, guilt, or worry [61], as a consequence of their excessive concern to satisfy their own and significant others' demands, the fear of failure or the guilt and shame that is usually experienced when they fail to reach their high standards. According to the results of this study, perfectionists would be characterized by excessively attending to their emotional responses. However, this excessive attention that perfectionists pay to their emotions (commonly negative) could develop into an emotional spiral, generating a ruminative process out of control, which, rather than alleviating their negative emotional state, would maintain it [53, 60]. In fact, the predisposition to rumination is a common characteristic of perfectionists [62].

### 4.3. Limitations and Future Research

Several limitations of the current study need to be mentioned. First, it is not possible to establish causality relationships due to the cross-sectional design. In this sense, using longitudinal and experimental designs may help explain the relationship (and its direction) between EI and perfectionism. In addition, it is important to underline that our participants were based on a normal sample of high-school students from Ecuador. So, these results should be generalized with caution to other samples such as clinical population or different ethnic groups. Additionally, it is necessary to mention that this study employs the SOP and SPP subscales as indicators of PS and PC. In this sense, it should be considered that some studies support the idea that SOP is better conceptualized using two dimensions, one maladaptive (SOP-Critical) and the other adaptive (SOP-Strivings) [63]. Although this issue is still under discussion, it would be interesting replicate this study analyzing these two dimensions separately. Moreover, because several subscales of perfectionism could be employed with this aim [2], it would be interesting to replicate this study using other measures of perfectionism, such as the Adaptive Maladaptive Perfectionism Scale (AMPS) [64] or the Multidimensional Perfectionism Scale (FMPS) [65]. Moreover, since TMMS is focused on intrapersonal dimensions of trait EI, future studies might use measures that include both intrapersonal and interpersonal subcomponents, as well as EI ability tests, such as The Mayer-Salovey-Caruso emotional intelligence test (MSCEIT) [66]. Finally, because gender differences have been observed in EI [67], future research should investigate if the results obtained in this study might be replicated when females and males are examined independently.

### 4.4. Conclusions and Practical Implications

Despite the limitations, this study is a novel contribution to perfectionism research as it provides both empirical and theoretical support for a three-profile classification of individuals according to their PS and PC scores (high, moderate, or low). In accordance with this three-class model, two out of ten teenagers, approximately, would present a high-risk profile of perfectionism. Moreover, this study also contributes to enriching the limited previous research about the relationship between perfectionism and EI. In this respect, this study evidences that there is a close relationship between perfectionism and the Emotional Attention component of EI which would be explained by both PS and PC perfectionistic dimensions. Thus, high perfectionists (individuals with high scores on both PS and PC) tend to score significantly higher on emotional attention in comparison with those individuals who were classified on the moderate and nonperfectionism profiles. As mentioned above, this tendency of high perfectionists to pay excessive attention to their own emotions (usually negative), far from being adaptive, might lead them to an out-of-control ruminative process.

The findings of this study have important practical implications for the treatment of perfectionism. Specifically, part of the therapeutic efforts should be devoted to intervention at the emotional level with the aim of preventing the appearance of maladaptive regulatory strategies such as rumination, which have been shown to predispose towards the development of psychopathologies such as depression [62]. In this sense, the main areas of action should be aimed at promoting the awareness of own emotions, but without giving an excessive self-focused attention, understanding own emotional states and replacing problematic regulatory strategies, such as rumination, by more effective ones, such as cognitive reappraisal or seeking social support [54].

## Figures and Tables

**Table 1:** Reliability, means, standard deviations, and bivariate and partial correlations between the factors of the CAPS and TMMS-24.

	Bivariate correlations		Partial correlations		*α*	*M*	*SD*
	*PS*	*PC*	*PS*	*PC*			
Emotional attention	0.18^*∗∗*^	0.16^*∗∗*^	0.11^*∗∗*^	0.08^*∗∗*^	0.87	15.70	6.83
Emotional clarity	0.08^*∗∗*^	0.04	0.07^*∗*^	0.01	0.80	16.84	6.34
Mood repair	0.11^*∗∗*^	0.04	0.11^*∗∗*^	−0.02	0.83	20.98	6.68
*α*	0.71	0.75	—	—	—	—	—
*M*	39.43	37.87	—	—	—	—	—
*SD*	6.46	6.75	—	—	—	—	—

*Note.* PS, perfectionistic standards, PC, perfectionistic concerns. ^*∗*^*p* < .05. ^*∗∗*^*p* < .01. ^*∗∗∗*^*p* < .001.

**Table 2:** Fit indexes of the results of the LCA.

Account of classes	*BIC*	Entropy
2 classes	8558.30	0.54
3 classes	8464.88	0.59
4 classes	8452.51	0.59
5 classes	8468.99	0.55
6 classes	8493.39	0.57

**Table 3:** Mean scores, standard deviations, and post-hoc contrasts obtained by the perfectionist profiles on EI sub-components.

EI sub-component	Moderate perfectionism		High perfectionism		Non-perfectionism		Statistical significance and effect sizes		
	*M*	*SD*	*M*	*SD*	*M*	*SD*	*F*_*(2.1579)*_	*p*	*η* [2]
Emotional attention	15.57	6.58	17.32	7.07	12.60	6.74	27.88	<0.001	0.034
Emotional clarity	16.61	6.15	17.62	6.67	16.50	6.55	3.79	0.023	0.005
Mood repair	20.67	6.55	21.85	6.75	20.83	7.20	4.45	0.012	0.006

**Table 4:** Cohen's d indexes for post-hoc contrasts between the mean scores obtained by the three classes on EI sub-components.

	Moderate perfectionism vs High perfectionism	Moderate perfectionism vs Non-perfectionism	High perfectionism vs Non-perfectionism
Emotional attention	0.26	0.45	0.68
Emotional clarity	0.16	—	—
Mood repair	0.18	—	—

## Data Availability

The datasets used and/or analyzed during the current study are available in the Supplementary Material.

## Supplementary Materials

Supplementary Materials The supplementary material includes the dataset about the sex, age, direct score on the three EI dimensions, and the perfectionism class in which each participant has been classified.
