# Supplementary material for: Perfectionism and Emotional Intelligence: A Person-Centered Approach
Source: Int J Clin Pract. 2022 Oct 29;2022:8660575. doi: 10.1155/2022/8660575 (PMC9637030; doi:10.1155/2022/8660575)
Supplement: Supplementary Materials — The supplementary material includes the dataset about the sex, age, direct score on the three EI dimensions, and the perfectionism class in which each participant has been classified. [file 8660575.f1.doc]

| *N* | Age | Sex | Emotional Attention | Emotional Clarity | Mood Repair | Class |
| --- | --- | --- | --- | --- | --- | --- |
|  | 12,0 | 2 | 15,00 | 15,00 | 14,00 | 1,00 |
|  | 12,0 | 1 | 31,00 | 28,00 | 28,00 | 1,00 |
|  | 12,0 | 1 | 17,00 | 14,00 | 25,00 | 1,00 |
|  | 13,0 | 1 | 20,00 | 16,00 | 21,00 | 1,00 |
|  | 12,0 | 1 | 8,00 | 12,00 | 19,00 | 1,00 |
|  | 12,0 | 2 | 18,00 | 21,00 | 26,00 | 1,00 |
|  | 12,0 | 2 | 30,00 | 32,00 | 32,00 | 2,00 |
|  | 12,0 | 1 | 13,00 | 10,00 | 22,00 | 3,00 |
|  | 13,0 | 2 | 19,00 | 19,00 | 25,00 | 1,00 |
|  | 12,0 | 2 | 32,00 | 32,00 | 32,00 | 1,00 |
|  | 12,0 | 2 | 17,00 | 15,00 | 22,00 | 1,00 |
|  | 12,0 | 1 | 21,00 | 11,00 | 17,00 | 1,00 |
|  | 13,0 | 1 | 15,00 | 21,00 | 17,00 | 1,00 |
|  | 13,0 | 2 | 24,00 | 21,00 | 29,00 | 1,00 |
|  | 13,0 | 1 | 11,00 | 16,00 | 18,00 | 1,00 |
|  | 14,0 | 1 | 12,00 | 16,00 | 15,00 | 1,00 |
|  | 13,0 | 2 | 19,00 | 30,00 | 25,00 | 2,00 |
|  | 13,0 | 1 | 14,00 | 8,00 | 22,00 | 2,00 |
|  | 13,0 | 2 | 3,00 | 7,00 | 12,00 | 1,00 |
|  | 12,0 | 1 | 7,00 | 11,00 | 14,00 | 2,00 |
|  | 12,0 | 1 | 26,00 | 21,00 | 22,00 | 2,00 |
|  | 13,0 | 1 | 26,00 | 17,00 | 14,00 | 2,00 |
|  | 13,0 | 1 | 15,00 | 19,00 | 15,00 | 1,00 |
|  | 12,0 | 1 | 23,00 | 22,00 | 26,00 | 1,00 |
|  | 13,0 | 2 | 19,00 | 17,00 | 16,00 | 2,00 |
|  | 13,0 | 1 | 22,00 | 21,00 | 25,00 | 1,00 |
|  | 14,0 | 1 | 16,00 | 16,00 | 16,00 | 1,00 |
|  | 12,0 | 2 | 23,00 | 19,00 | 24,00 | 2,00 |
|  | 14,0 | 2 | 25,00 | 16,00 | 21,00 | 1,00 |
|  | 12,0 | 1 | 18,00 | 13,00 | 21,00 | 1,00 |
|  | 12,0 | 1 | 11,00 | 15,00 | 19,00 | 2,00 |
|  | 12,0 | 2 | 27,00 | 19,00 | 31,00 | 2,00 |
|  | 12,0 | 1 | 4,00 | 3,00 | 7,00 | 1,00 |
|  | 15,0 | 1 | 8,00 | 9,00 | 10,00 | 3,00 |
|  | 12,0 | 1 | 20,00 | 23,00 | 29,00 | 2,00 |
|  | 12,0 | 1 | 14,00 | 28,00 | 28,00 | 1,00 |
|  | 13,0 | 1 | 18,00 | 18,00 | 23,00 | 1,00 |
|  | 13,0 | 2 | 15,00 | 21,00 | 31,00 | 1,00 |
|  | 12,0 | 1 | 21,00 | 24,00 | 27,00 | 1,00 |
|  | 13,0 | 1 | 19,00 | 20,00 | 19,00 | 1,00 |
|  | 12,0 | 1 | 15,00 | 20,00 | 22,00 | 1,00 |
|  | 13,0 | 1 | 24,00 | 25,00 | 26,00 | 1,00 |
|  | 13,0 | 1 | 25,00 | 14,00 | 19,00 | 1,00 |
|  | 12,0 | 2 | 25,00 | 15,00 | 32,00 | 1,00 |
|  | 12,0 | 1 | 24,00 | 23,00 | 28,00 | 2,00 |
|  | 13,0 | 1 | 23,00 | 29,00 | 22,00 | 2,00 |
|  | 12,0 | 2 | 16,00 | 13,00 | 22,00 | 2,00 |
|  | 13,0 | 2 | 14,00 | 22,00 | 13,00 | 3,00 |
|  | 12,0 | 2 | 4,00 | 5,00 | 10,00 | 3,00 |
|  | 12,0 | 2 | 12,00 | 27,00 | 26,00 | 3,00 |
|  | 13,0 | 1 | 27,00 | 24,00 | 17,00 | 2,00 |
|  | 12,0 | 2 | 10,00 | 13,00 | 12,00 | 1,00 |
|  | 14,0 | 1 | 19,00 | 7,00 | 14,00 | 1,00 |
|  | 12,0 | 2 | 15,00 | 10,00 | 15,00 | 1,00 |
|  | 13,0 | 1 | 28,00 | 15,00 | 18,00 | 1,00 |
|  | 12,0 | 1 | 9,00 | 13,00 | 16,00 | 1,00 |
|  | 13,0 | 2 | 32,00 | 21,00 | 17,00 | 2,00 |
|  | 13,0 | 2 | 12,00 | 18,00 | 26,00 | 3,00 |
|  | 13,0 | 1 | 12,00 | 31,00 | 26,00 | 1,00 |
|  | 14,0 | 2 | 19,00 | 16,00 | 25,00 | 1,00 |
|  | 13,0 | 1 | 21,00 | 20,00 | 20,00 | 1,00 |
|  | 12,0 | 1 | 20,00 | 18,00 | 17,00 | 1,00 |
|  | 13,0 | 1 | 14,00 | 15,00 | 17,00 | 1,00 |
|  | 12,0 | 2 | 28,00 | 27,00 | 18,00 | 2,00 |
|  | 12,0 | 1 | 16,00 | 17,00 | 8,00 | 1,00 |
|  | 12,0 | 2 | 27,00 | 28,00 | 27,00 | 2,00 |
|  | 12,0 | 2 | 12,00 | 15,00 | 17,00 | 1,00 |
|  | 12,0 | 1 | 27,00 | 22,00 | 23,00 | 1,00 |
|  | 12,0 | 1 | 22,00 | 31,00 | 24,00 | 1,00 |
|  | 12,0 | 1 | 18,00 | 24,00 | 20,00 | 1,00 |
|  | 13,0 | 2 | 17,00 | 21,00 | 30,00 | 2,00 |
|  | 13,0 | 1 | 29,00 | 10,00 | 31,00 | 2,00 |
|  | 12,0 | 1 | 15,00 | 18,00 | 21,00 | 1,00 |
|  | 12,0 | 1 | 11,00 | 19,00 | 18,00 | 1,00 |
|  | 13,0 | 2 | 9,00 | 14,00 | 24,00 | 1,00 |
|  | 12,0 | 1 | 6,00 | 12,00 | 11,00 | 1,00 |
|  | 12,0 | 2 | 17,00 | 19,00 | 22,00 | 1,00 |
|  | 12,0 | 1 | 15,00 | 15,00 | 23,00 | 1,00 |
|  | 13,0 | 2 | 21,00 | 21,00 | 23,00 | 1,00 |
|  | 12,0 | 1 | 26,00 | 21,00 | 23,00 | 2,00 |
|  | 13,0 | 2 | 8,00 | 24,00 | 28,00 | 2,00 |
|  | 12,0 | 2 | 30,00 | 16,00 | 30,00 | 2,00 |
|  | 12,0 | 2 | 16,00 | 25,00 | 23,00 | 2,00 |
|  | 13,0 | 1 | 17,00 | 20,00 | 24,00 | 1,00 |
|  | 12,0 | 2 | 16,00 | 22,00 | 28,00 | 1,00 |
|  | 14,0 | 1 | 9,00 | 9,00 | 11,00 | 2,00 |
|  | 12,0 | 2 | 7,00 | 14,00 | 17,00 | 3,00 |
|  | 13,0 | 1 | 15,00 | 17,00 | 21,00 | 2,00 |
|  | 13,0 | 1 | 3,00 | 11,00 | 19,00 | 1,00 |
|  | 13,0 | 2 | 23,00 | 24,00 | 27,00 | 2,00 |
|  | 12,0 | 1 | 22,00 | 9,00 | 22,00 | 1,00 |
|  | 12,0 | 1 | 19,00 | 19,00 | 18,00 | 1,00 |
|  | 12,0 | 1 | 19,00 | 16,00 | 15,00 | 1,00 |
|  | 12,0 | 2 | 18,00 | 17,00 | 16,00 | 1,00 |
|  | 12,0 | 2 | 21,00 | 10,00 | 14,00 | 1,00 |
|  | 13,0 | 1 | 21,00 | 19,00 | 24,00 | 1,00 |
|  | 13,0 | 1 | 9,00 | 8,00 | 15,00 | 1,00 |
|  | 12,0 | 1 | 28,00 | 25,00 | 26,00 | 1,00 |
|  | 12,0 | 1 | 12,00 | 6,00 | 14,00 | 1,00 |
|  | 14,0 | 2 | 23,00 | 14,00 | 14,00 | 1,00 |
|  | 12,0 | 2 | 12,00 | 18,00 | 16,00 | 1,00 |
|  | 14,0 | 1 | 14,00 | 22,00 | 21,00 | 1,00 |
|  | 13,0 | 1 | 24,00 | 10,00 | 19,00 | 2,00 |
|  | 14,0 | 1 | 25,00 | 32,00 | 24,00 | 2,00 |
|  | 12,0 | 1 | 9,00 | 20,00 | 30,00 | 1,00 |
|  | 13,0 | 1 | 14,00 | 15,00 | 21,00 | 2,00 |
|  | 13,0 | 2 | 16,00 | 23,00 | 25,00 | 3,00 |
|  | 13,0 | 1 | 14,00 | 11,00 | 10,00 | 2,00 |
|  | 14,0 | 1 | 18,00 | 19,00 | 23,00 | 2,00 |
|  | 14,0 | 1 | 19,00 | 10,00 | 19,00 | 2,00 |
|  | 14,0 | 1 | 15,00 | 12,00 | 21,00 | 1,00 |
|  | 13,0 | 2 | 15,00 | 14,00 | 23,00 | 1,00 |
|  | 14,0 | 1 | 32,00 | 32,00 | 32,00 | 2,00 |
|  | 15,0 | 2 | 8,00 | 18,00 | 9,00 | 2,00 |
|  | 12,0 | 1 | ,00 | 2,00 | 3,00 | 1,00 |
|  | 13,0 | 1 | 14,00 | 19,00 | 27,00 | 1,00 |
|  | 13,0 | 1 | 19,00 | 24,00 | 22,00 | 1,00 |
|  | 14,0 | 1 | 10,00 | 9,00 | 8,00 | 1,00 |
|  | 14,0 | 2 | 7,00 | 4,00 | 21,00 | 1,00 |
|  | 14,0 | 2 | 26,00 | 22,00 | 22,00 | 2,00 |
|  | 13,0 | 2 | 23,00 | 26,00 | 18,00 | 1,00 |
|  | 13,0 | 1 | 21,00 | 21,00 | 28,00 | 2,00 |
|  | 13,0 | 2 | 18,00 | 17,00 | 25,00 | 1,00 |
|  | 14,0 | 1 | 17,00 | 17,00 | 23,00 | 2,00 |
|  | 14,0 | 2 | 27,00 | 29,00 | 23,00 | 2,00 |
|  | 13,0 | 2 | 9,00 | 19,00 | 12,00 | 1,00 |
|  | 13,0 | 2 | 10,00 | 18,00 | 22,00 | 1,00 |
|  | 13,0 | 1 | 20,00 | 16,00 | 18,00 | 1,00 |
|  | 13,0 | 2 | 20,00 | 14,00 | 26,00 | 2,00 |
|  | 14,0 | 2 | 26,00 | 21,00 | 26,00 | 2,00 |
|  | 13,0 | 1 | 13,00 | 22,00 | 14,00 | 3,00 |
|  | 13,0 | 2 | 20,00 | 20,00 | 28,00 | 2,00 |
|  | 13,0 | 2 | 22,00 | 16,00 | 16,00 | 2,00 |
|  | 14,0 | 1 | 25,00 | 26,00 | 24,00 | 1,00 |
|  | 13,0 | 1 | 12,00 | 27,00 | 32,00 | 3,00 |
|  | 13,0 | 2 | 13,00 | 12,00 | 17,00 | 1,00 |
|  | 13,0 | 1 | 21,00 | 13,00 | 19,00 | 1,00 |
|  | 13,0 | 2 | 12,00 | 1,00 | ,00 | 2,00 |
|  | 13,0 | 1 | 14,00 | 21,00 | 20,00 | 2,00 |
|  | 13,0 | 2 | 17,00 | 29,00 | 29,00 | 1,00 |
|  | 13,0 | 1 | 28,00 | 28,00 | 24,00 | 2,00 |
|  | 14,0 | 2 | 11,00 | 15,00 | 14,00 | 2,00 |
|  | 14,0 | 2 | 19,00 | 18,00 | 24,00 | 1,00 |
|  | 14,0 | 1 | 9,00 | 12,00 | 12,00 | 2,00 |
|  | 13,0 | 1 | 16,00 | 14,00 | 27,00 | 2,00 |
|  | 13,0 | 1 | 9,00 | 18,00 | 21,00 | 1,00 |
|  | 13,0 | 1 | 25,00 | 17,00 | 24,00 | 2,00 |
|  | 14,0 | 1 | 13,00 | 15,00 | 16,00 | 2,00 |
|  | 14,0 | 1 | 18,00 | 21,00 | 22,00 | 1,00 |
|  | 14,0 | 1 | 4,00 | 24,00 | 24,00 | 2,00 |
|  | 13,0 | 1 | 32,00 | 20,00 | 12,00 | 1,00 |
|  | 13,0 | 1 | 20,00 | 14,00 | 18,00 | 1,00 |
|  | 14,0 | 2 | 17,00 | 31,00 | 32,00 | 3,00 |
|  | 14,0 | 2 | 17,00 | 24,00 | 30,00 | 1,00 |
|  | 13,0 | 2 | 19,00 | 22,00 | 32,00 | 1,00 |
|  | 13,0 | 1 | 25,00 | 21,00 | 29,00 | 2,00 |
|  | 13,0 | 2 | 7,00 | 6,00 | 19,00 | 3,00 |
|  | 14,0 | 1 | 18,00 | 19,00 | 15,00 | 2,00 |
|  | 13,0 | 1 | 13,00 | 14,00 | 11,00 | 1,00 |
|  | 13,0 | 2 | 11,00 | 18,00 | 20,00 | 2,00 |
|  | 13,0 | 1 | 10,00 | 13,00 | 13,00 | 1,00 |
|  | 13,0 | 2 | 11,00 | 11,00 | 20,00 | 1,00 |
|  | 14,0 | 1 | 2,00 | 7,00 | 8,00 | 3,00 |
|  | 13,0 | 1 | 6,00 | 7,00 | 18,00 | 3,00 |
|  | 13,0 | 1 | 20,00 | 15,00 | 14,00 | 2,00 |
|  | 13,0 | 1 | 18,00 | 18,00 | 14,00 | 1,00 |
|  | 13,0 | 2 | 23,00 | 26,00 | 26,00 | 1,00 |
|  | 13,0 | 2 | 15,00 | 18,00 | 25,00 | 1,00 |
|  | 14,0 | 1 | 23,00 | 26,00 | 28,00 | 2,00 |
|  | 14,0 | 1 | 22,00 | 21,00 | 30,00 | 2,00 |
|  | 13,0 | 1 | 6,00 | 11,00 | 16,00 | 2,00 |
|  | 13,0 | 1 | 18,00 | 18,00 | 23,00 | 2,00 |
|  | 15,0 | 2 | 9,00 | 12,00 | 28,00 | 1,00 |
|  | 13,0 | 1 | 14,00 | 18,00 | 20,00 | 1,00 |
|  | 13,0 | 1 | 17,00 | 15,00 | 19,00 | 1,00 |
|  | 13,0 | 2 | 8,00 | 7,00 | 23,00 | 1,00 |
|  | 13,0 | 2 | 9,00 | 17,00 | 29,00 | 1,00 |
|  | 13,0 | 2 | 11,00 | 23,00 | 24,00 | 2,00 |
|  | 14,0 | 2 | 13,00 | 16,00 | 17,00 | 1,00 |
|  | 13,0 | 2 | 19,00 | 31,00 | 32,00 | 2,00 |
|  | 13,0 | 2 | 17,00 | 16,00 | 24,00 | 2,00 |
|  | 13,0 | 2 | 10,00 | 14,00 | 29,00 | 2,00 |
|  | 13,0 | 1 | 13,00 | 9,00 | 21,00 | 2,00 |
|  | 13,0 | 1 | 11,00 | 11,00 | 10,00 | 1,00 |
|  | 13,0 | 2 | 20,00 | 20,00 | 28,00 | 1,00 |
|  | 13,0 | 1 | 18,00 | 18,00 | 31,00 | 1,00 |
|  | 13,0 | 1 | 19,00 | 14,00 | 9,00 | 2,00 |
|  | 13,0 | 1 | 20,00 | 22,00 | 24,00 | 1,00 |
|  | 13,0 | 1 | 8,00 | 12,00 | 21,00 | 1,00 |
|  | 13,0 | 1 | 20,00 | 17,00 | 16,00 | 1,00 |
|  | 13,0 | 2 | 16,00 | 17,00 | 21,00 | 2,00 |
|  | 13,0 | 1 | 6,00 | 17,00 | 22,00 | 2,00 |
|  | 13,0 | 1 | 5,00 | 7,00 | 4,00 | 3,00 |
|  | 13,0 | 1 | 12,00 | 22,00 | 20,00 | 1,00 |
|  | 13,0 | 1 | 4,00 | 5,00 | 4,00 | 3,00 |
|  | 13,0 | 1 | 26,00 | 22,00 | 21,00 | 2,00 |
|  | 13,0 | 2 | 13,00 | 9,00 | 8,00 | 1,00 |
|  | 13,0 | 2 | 10,00 | 19,00 | 30,00 | 1,00 |
|  | 13,0 | 1 | 13,00 | 12,00 | 14,00 | 1,00 |
|  | 13,0 | 2 | 11,00 | 16,00 | 10,00 | 1,00 |
|  | 13,0 | 1 | 3,00 | 18,00 | 13,00 | 1,00 |
|  | 13,0 | 1 | 13,00 | 16,00 | 27,00 | 2,00 |
|  | 13,0 | 2 | 14,00 | 19,00 | 22,00 | 1,00 |
|  | 13,0 | 1 | 20,00 | 25,00 | 21,00 | 2,00 |
|  | 13,0 | 2 | 10,00 | 10,00 | 14,00 | 2,00 |
|  | 13,0 | 2 | 28,00 | 15,00 | 28,00 | 1,00 |
|  | 13,0 | 1 | 8,00 | 7,00 | 10,00 | 2,00 |
|  | 13,0 | 1 | 13,00 | 18,00 | 24,00 | 1,00 |
|  | 13,0 | 1 | 2,00 | 31,00 | 8,00 | 2,00 |
|  | 14,0 | 1 | 19,00 | 23,00 | 27,00 | 1,00 |
|  | 13,0 | 1 | 20,00 | 13,00 | 20,00 | 3,00 |
|  | 14,0 | 1 | 30,00 | 26,00 | 26,00 | 2,00 |
|  | 13,0 | 2 | ,00 | ,00 | ,00 | 2,00 |
|  | 13,0 | 1 | 12,00 | 17,00 | 16,00 | 1,00 |
|  | 13,0 | 1 | 32,00 | 32,00 | 32,00 | 2,00 |
|  | 13,0 | 2 | 26,00 | 28,00 | 27,00 | 1,00 |
|  | 14,0 | 2 | 16,00 | 20,00 | 28,00 | 1,00 |
|  | 13,0 | 1 | 11,00 | 19,00 | 21,00 | 1,00 |
|  | 14,0 | 1 | 10,00 | 15,00 | 16,00 | 2,00 |
|  | 13,0 | 1 | 26,00 | 27,00 | 24,00 | 1,00 |
|  | 13,0 | 1 | 17,00 | 19,00 | 11,00 | 2,00 |
|  | 13,0 | 1 | 25,00 | 20,00 | 26,00 | 2,00 |
|  | 13,0 | 1 | 23,00 | 29,00 | 21,00 | 1,00 |
|  | 13,0 | 2 | 16,00 | 8,00 | 25,00 | 1,00 |
|  | 13,0 | 2 | 10,00 | 16,00 | 21,00 | 1,00 |
|  | 13,0 | 1 | 18,00 | 11,00 | 21,00 | 2,00 |
|  | 13,0 | 1 | 11,00 | 18,00 | 26,00 | 1,00 |
|  | 13,0 | 2 | 13,00 | 22,00 | 22,00 | 1,00 |
|  | 13,0 | 1 | 20,00 | 25,00 | 28,00 | 2,00 |
|  | 14,0 | 2 | 24,00 | 15,00 | 30,00 | 2,00 |
|  | 14,0 | 2 | 28,00 | 9,00 | 14,00 | 2,00 |
|  | 14,0 | 2 | 6,00 | 11,00 | 24,00 | 1,00 |
|  | 13,0 | 1 | 15,00 | 18,00 | 24,00 | 1,00 |
|  | 13,0 | 2 | 17,00 | 19,00 | 17,00 | 1,00 |
|  | 14,0 | 1 | 17,00 | 15,00 | 20,00 | 1,00 |
|  | 14,0 | 1 | 9,00 | 19,00 | 19,00 | 1,00 |
|  | 13,0 | 2 | 12,00 | 24,00 | 29,00 | 1,00 |
|  | 14,0 | 2 | 13,00 | 18,00 | 26,00 | 1,00 |
|  | 13,0 | 1 | 5,00 | 21,00 | ,00 | 1,00 |
|  | 13,0 | 2 | 22,00 | 21,00 | 7,00 | 1,00 |
|  | 14,0 | 1 | 15,00 | 17,00 | 17,00 | 1,00 |
|  | 14,0 | 1 | 14,00 | 15,00 | 26,00 | 1,00 |
|  | 14,0 | 2 | 10,00 | 13,00 | 20,00 | 2,00 |
|  | 13,0 | 1 | 13,00 | 24,00 | 25,00 | 1,00 |
|  | 14,0 | 1 | 13,00 | 11,00 | 18,00 | 1,00 |
|  | 13,0 | 1 | 15,00 | 14,00 | 14,00 | 1,00 |
|  | 14,0 | 2 | 12,00 | 19,00 | 16,00 | 1,00 |
|  | 15,0 | 2 | 21,00 | 6,00 | 25,00 | 1,00 |
|  | 14,0 | 1 | 20,00 | 24,00 | 20,00 | 3,00 |
|  | 15,0 | 1 | 26,00 | 9,00 | 11,00 | 1,00 |
|  | 14,0 | 2 | 10,00 | 12,00 | 12,00 | 1,00 |
|  | 14,0 | 1 | 8,00 | 18,00 | 28,00 | 3,00 |
|  | 14,0 | 2 | 6,00 | 17,00 | 28,00 | 2,00 |
|  | 15,0 | 2 | 9,00 | 9,00 | 9,00 | 1,00 |
|  | 14,0 | 2 | 20,00 | 20,00 | 30,00 | 1,00 |
|  | 15,0 | 2 | 8,00 | 5,00 | 8,00 | 1,00 |
|  | 14,0 | 1 | 17,00 | 19,00 | 17,00 | 1,00 |
|  | 14,0 | 1 | 12,00 | 7,00 | 13,00 | 1,00 |
|  | 15,0 | 1 | 25,00 | 21,00 | 23,00 | 1,00 |
|  | 14,0 | 2 | 14,00 | 11,00 | 26,00 | 1,00 |
|  | 14,0 | 1 | 19,00 | 24,00 | 26,00 | 1,00 |
|  | 14,0 | 2 | 11,00 | 6,00 | 13,00 | 1,00 |
|  | 15,0 | 1 | 26,00 | 17,00 | 16,00 | 2,00 |
|  | 14,0 | 2 | 5,00 | 11,00 | 15,00 | 1,00 |
|  | 14,0 | 2 | 22,00 | 32,00 | 31,00 | 2,00 |
|  | 16,0 | 1 | 3,00 | 2,00 | 4,00 | 3,00 |
|  | 14,0 | 2 | 12,00 | 10,00 | 28,00 | 1,00 |
|  | 14,0 | 2 | 13,00 | 9,00 | 13,00 | 3,00 |
|  | 15,0 | 1 | 11,00 | 18,00 | 30,00 | 2,00 |
|  | 14,0 | 2 | 21,00 | 21,00 | 20,00 | 1,00 |
|  | 15,0 | 1 | 12,00 | 21,00 | 23,00 | 3,00 |
|  | 15,0 | 1 | 17,00 | 24,00 | 22,00 | 1,00 |
|  | 14,0 | 2 | 11,00 | 5,00 | 8,00 | 1,00 |
|  | 14,0 | 1 | 18,00 | 21,00 | 23,00 | 2,00 |
|  | 15,0 | 1 | 24,00 | 32,00 | 30,00 | 2,00 |
|  | 15,0 | 1 | 14,00 | 15,00 | 27,00 | 2,00 |
|  | 14,0 | 2 | 17,00 | 17,00 | 19,00 | 1,00 |
|  | 15,0 | 1 | 21,00 | 25,00 | 25,00 | 2,00 |
|  | 14,0 | 1 | 15,00 | 10,00 | 21,00 | 1,00 |
|  | 15,0 | 1 | 13,00 | 21,00 | 26,00 | 1,00 |
|  | 15,0 | 1 | 18,00 | 19,00 | 25,00 | 1,00 |
|  | 14,0 | 1 | 11,00 | 9,00 | 21,00 | 3,00 |
|  | 16,0 | 1 | 22,00 | 30,00 | 26,00 | 2,00 |
|  | 14,0 | 1 | 16,00 | 15,00 | 19,00 | 1,00 |
|  | 14,0 | 1 | 13,00 | 20,00 | 28,00 | 1,00 |
|  | 15,0 | 1 | 16,00 | 12,00 | 16,00 | 2,00 |
|  | 14,0 | 2 | 31,00 | 28,00 | 6,00 | 2,00 |
|  | 14,0 | 1 | 15,00 | 19,00 | 16,00 | 1,00 |
|  | 14,0 | 1 | 15,00 | 20,00 | 29,00 | 1,00 |
|  | 15,0 | 1 | 16,00 | 20,00 | 21,00 | 2,00 |
|  | 14,0 | 1 | 28,00 | 32,00 | 32,00 | 1,00 |
|  | 16,0 | 1 | 8,00 | 16,00 | 17,00 | 1,00 |
|  | 15,0 | 2 | 6,00 | 13,00 | 21,00 | 1,00 |
|  | 15,0 | 1 | 5,00 | 10,00 | 18,00 | 1,00 |
|  | 14,0 | 2 | 6,00 | 10,00 | 16,00 | 1,00 |
|  | 14,0 | 1 | 9,00 | 12,00 | 20,00 | 1,00 |
|  | 16,0 | 1 | 8,00 | 26,00 | 29,00 | 2,00 |
|  | 14,0 | 1 | 13,00 | 13,00 | 18,00 | 1,00 |
|  | 14,0 | 1 | 20,00 | 16,00 | 18,00 | 1,00 |
|  | 14,0 | 1 | 20,00 | 17,00 | 23,00 | 1,00 |
|  | 14,0 | 1 | 18,00 | 11,00 | 5,00 | 1,00 |
|  | 14,0 | 2 | 11,00 | 13,00 | 29,00 | 1,00 |
|  | 14,0 | 2 | 5,00 | 10,00 | 20,00 | 1,00 |
|  | 15,0 | 1 | 3,00 | 8,00 | 1,00 | 1,00 |
|  | 14,0 | 1 | 10,00 | 17,00 | 21,00 | 1,00 |
|  | 15,0 | 1 | 14,00 | 11,00 | 24,00 | 2,00 |
|  | 15,0 | 2 | 6,00 | 32,00 | 28,00 | 2,00 |
|  | 15,0 | 1 | 16,00 | 12,00 | 26,00 | 2,00 |
|  | 14,0 | 2 | 18,00 | 9,00 | 15,00 | 1,00 |
|  | 15,0 | 2 | 13,00 | 10,00 | 16,00 | 2,00 |
|  | 14,0 | 1 | 17,00 | 22,00 | 24,00 | 1,00 |
|  | 14,0 | 1 | 24,00 | 24,00 | 28,00 | 1,00 |
|  | 15,0 | 1 | 6,00 | 11,00 | 17,00 | 3,00 |
|  | 14,0 | 1 | 8,00 | 19,00 | 30,00 | 1,00 |
|  | 14,0 | 2 | 10,00 | 17,00 | 23,00 | 1,00 |
|  | 14,0 | 1 | 6,00 | 8,00 | 29,00 | 2,00 |
|  | 14,0 | 2 | 11,00 | 20,00 | 18,00 | 1,00 |
|  | 14,0 | 1 | 13,00 | 14,00 | 18,00 | 1,00 |
|  | 14,0 | 2 | 11,00 | 13,00 | 27,00 | 1,00 |
|  | 15,0 | 1 | 26,00 | 22,00 | 26,00 | 1,00 |
|  | 15,0 | 1 | 17,00 | 20,00 | 11,00 | 2,00 |
|  | 15,0 | 1 | 20,00 | 16,00 | 23,00 | 1,00 |
|  | 14,0 | 1 | 10,00 | 9,00 | 9,00 | 1,00 |
|  | 14,0 | 2 | 4,00 | 17,00 | 25,00 | 1,00 |
|  | 15,0 | 2 | 9,00 | 14,00 | 23,00 | 2,00 |
|  | 14,0 | 1 | 17,00 | 14,00 | 24,00 | 1,00 |
|  | 14,0 | 1 | 8,00 | 15,00 | 15,00 | 2,00 |
|  | 14,0 | 1 | 19,00 | 18,00 | 21,00 | 1,00 |
|  | 15,0 | 1 | 26,00 | 24,00 | 25,00 | 1,00 |
|  | 14,0 | 1 | 17,00 | 16,00 | 18,00 | 2,00 |
|  | 17,0 | 1 | 27,00 | 27,00 | 28,00 | 1,00 |
|  | 15,0 | 2 | 32,00 | 22,00 | 26,00 | 1,00 |
|  | 15,0 | 1 | 18,00 | 20,00 | 21,00 | 1,00 |
|  | 15,0 | 1 | 7,00 | 7,00 | 29,00 | 2,00 |
|  | 14,0 | 1 | 15,00 | 17,00 | 15,00 | 1,00 |
|  | 14,0 | 2 | 20,00 | 8,00 | 26,00 | 2,00 |
|  | 14,0 | 2 | 26,00 | 10,00 | 13,00 | 1,00 |
|  | 14,0 | 2 | 19,00 | 13,00 | 28,00 | 1,00 |
|  | 15,0 | 2 | 10,00 | 5,00 | 11,00 | 1,00 |
|  | 14,0 | 2 | 12,00 | 32,00 | 20,00 | 2,00 |
|  | 14,0 | 1 | 10,00 | 17,00 | 21,00 | 1,00 |
|  | 15,0 | 2 | 23,00 | 24,00 | 24,00 | 2,00 |
|  | 15,0 | 1 | 15,00 | 16,00 | 13,00 | 1,00 |
|  | 14,0 | 1 | 18,00 | 16,00 | 29,00 | 2,00 |
|  | 15,0 | 1 | 20,00 | 16,00 | 21,00 | 2,00 |
|  | 14,0 | 1 | 14,00 | 13,00 | 23,00 | 1,00 |
|  | 14,0 | 1 | 8,00 | 20,00 | 21,00 | 3,00 |
|  | 15,0 | 2 | 17,00 | 14,00 | 20,00 | 1,00 |
|  | 14,0 | 2 | 18,00 | 25,00 | 31,00 | 1,00 |
|  | 14,0 | 1 | 4,00 | 10,00 | 4,00 | 1,00 |
|  | 14,0 | 2 | 27,00 | 29,00 | 32,00 | 1,00 |
|  | 14,0 | 1 | ,00 | 15,00 | 4,00 | 3,00 |
|  | 15,0 | 1 | 11,00 | 15,00 | 12,00 | 1,00 |
|  | 14,0 | 1 | 9,00 | 20,00 | 32,00 | 1,00 |
|  | 13,0 | 1 | 7,00 | 14,00 | 13,00 | 1,00 |
|  | 15,0 | 1 | 18,00 | 11,00 | 22,00 | 1,00 |
|  | 14,0 | 1 | 28,00 | 32,00 | 22,00 | 3,00 |
|  | 14,0 | 2 | 32,00 | 25,00 | 29,00 | 1,00 |
|  | 15,0 | 1 | 20,00 | 21,00 | 29,00 | 3,00 |
|  | 14,0 | 1 | 9,00 | 22,00 | 21,00 | 1,00 |
|  | 14,0 | 1 | 17,00 | 18,00 | 24,00 | 1,00 |
|  | 15,0 | 2 | 17,00 | 10,00 | 14,00 | 1,00 |
|  | 15,0 | 1 | 12,00 | 19,00 | 13,00 | 1,00 |
|  | 15,0 | 1 | 12,00 | 21,00 | 31,00 | 1,00 |
|  | 14,0 | 1 | 5,00 | 16,00 | 17,00 | 1,00 |
|  | 14,0 | 1 | 3,00 | 10,00 | 17,00 | 1,00 |
|  | 16,0 | 1 | 19,00 | 17,00 | 21,00 | 1,00 |
|  | 15,0 | 1 | 22,00 | 14,00 | 26,00 | 1,00 |
|  | 14,0 | 2 | 10,00 | 4,00 | 23,00 | 2,00 |
|  | 16,0 | 1 | 13,00 | 14,00 | 18,00 | 1,00 |
|  | 15,0 | 1 | 24,00 | 32,00 | 32,00 | 2,00 |
|  | 14,0 | 1 | 18,00 | 11,00 | 24,00 | 1,00 |
|  | 14,0 | 1 | 15,00 | 13,00 | 16,00 | 1,00 |
|  | 14,0 | 2 | 13,00 | 2,00 | 6,00 | 3,00 |
|  | 14,0 | 1 | 17,00 | 26,00 | 27,00 | 1,00 |
|  | 15,0 | 1 | 15,00 | 10,00 | 25,00 | 3,00 |
|  | 14,0 | 1 | 15,00 | 29,00 | 28,00 | 1,00 |
|  | 14,0 | 2 | 28,00 | 30,00 | 28,00 | 1,00 |
|  | 14,0 | 1 | 10,00 | 13,00 | 22,00 | 2,00 |
|  | 14,0 | 2 | 14,00 | 15,00 | 22,00 | 1,00 |
|  | 15,0 | 1 | 11,00 | 10,00 | 9,00 | 3,00 |
|  | 15,0 | 2 | 11,00 | 12,00 | 14,00 | 1,00 |
|  | 15,0 | 1 | 10,00 | 12,00 | 21,00 | 1,00 |
|  | 15,0 | 1 | 18,00 | 15,00 | 28,00 | 2,00 |
|  | 14,0 | 1 | 10,00 | 10,00 | 12,00 | 1,00 |
|  | 14,0 | 1 | 13,00 | 20,00 | 28,00 | 1,00 |
|  | 14,0 | 2 | 9,00 | 1,00 | 5,00 | 1,00 |
|  | 15,0 | 1 | 15,00 | 30,00 | 30,00 | 1,00 |
|  | 14,0 | 2 | 20,00 | 14,00 | 21,00 | 1,00 |
|  | 14,0 | 1 | 14,00 | 19,00 | 28,00 | 1,00 |
|  | 14,0 | 1 | 8,00 | 19,00 | 30,00 | 3,00 |
|  | 14,0 | 2 | 10,00 | 19,00 | 30,00 | 2,00 |
|  | 14,0 | 1 | 10,00 | 13,00 | 24,00 | 2,00 |
|  | 14,0 | 2 | 8,00 | 20,00 | 18,00 | 3,00 |
|  | 14,0 | 2 | 4,00 | 28,00 | 16,00 | 1,00 |
|  | 15,0 | 1 | 8,00 | 15,00 | 10,00 | 1,00 |
|  | 18,0 | 1 | 8,00 | 15,00 | 29,00 | 2,00 |
|  | 16,0 | 1 | 16,00 | 15,00 | 24,00 | 1,00 |
|  | 15,0 | 2 | 24,00 | 15,00 | 27,00 | 1,00 |
|  | 15,0 | 1 | 27,00 | 22,00 | 22,00 | 1,00 |
|  | 15,0 | 1 | 19,00 | 19,00 | 9,00 | 1,00 |
|  | 15,0 | 1 | 17,00 | 15,00 | 17,00 | 1,00 |
|  | 16,0 | 1 | 15,00 | 16,00 | 26,00 | 1,00 |
|  | 16,0 | 1 | 15,00 | 18,00 | 27,00 | 1,00 |
|  | 17,0 | 1 | 16,00 | 10,00 | 16,00 | 1,00 |
|  | 15,0 | 2 | 24,00 | 25,00 | 32,00 | 2,00 |
|  | 15,0 | 1 | 10,00 | 8,00 | 8,00 | 1,00 |
|  | 15,0 | 1 | 26,00 | 23,00 | 23,00 | 2,00 |
|  | 16,0 | 1 | 13,00 | 11,00 | 15,00 | 2,00 |
|  | 15,0 | 1 | 13,00 | 22,00 | 19,00 | 1,00 |
|  | 16,0 | 1 | 19,00 | 24,00 | 24,00 | 2,00 |
|  | 16,0 | 1 | 24,00 | 5,00 | 1,00 | 1,00 |
|  | 16,0 | 1 | 25,00 | 20,00 | 19,00 | 2,00 |
|  | 16,0 | 1 | 21,00 | 18,00 | 29,00 | 2,00 |
|  | 16,0 | 1 | 8,00 | 7,00 | 18,00 | 2,00 |
|  | 17,0 | 1 | 29,00 | 18,00 | 22,00 | 1,00 |
|  | 16,0 | 1 | 2,00 | 12,00 | 9,00 | 3,00 |
|  | 18,0 | 1 | 16,00 | 13,00 | 16,00 | 1,00 |
|  | 15,0 | 1 | 1,00 | 26,00 | 2,00 | 3,00 |
|  | 16,0 | 1 | 16,00 | 10,00 | 23,00 | 1,00 |
|  | 16,0 | 1 | 6,00 | 4,00 | 27,00 | 1,00 |
|  | 15,0 | 2 | 19,00 | 20,00 | 10,00 | 1,00 |
|  | 17,0 | 1 | 28,00 | 29,00 | 23,00 | 1,00 |
|  | 15,0 | 1 | 20,00 | 27,00 | 28,00 | 1,00 |
|  | 15,0 | 1 | 16,00 | 19,00 | 17,00 | 1,00 |
|  | 15,0 | 1 | 13,00 | 17,00 | 28,00 | 2,00 |
|  | 15,0 | 1 | 18,00 | 21,00 | 24,00 | 1,00 |
|  | 15,0 | 1 | 29,00 | 21,00 | 13,00 | 1,00 |
|  | 15,0 | 1 | 19,00 | 20,00 | 20,00 | 1,00 |
|  | 15,0 | 1 | 13,00 | 15,00 | 21,00 | 1,00 |
|  | 16,0 | 1 | 14,00 | 21,00 | 29,00 | 2,00 |
|  | 16,0 | 1 | 22,00 | 21,00 | 20,00 | 2,00 |
|  | 16,0 | 1 | 2,00 | 2,00 | 2,00 | 1,00 |
|  | 16,0 | 1 | 5,00 | 17,00 | 9,00 | 1,00 |
|  | 16,0 | 1 | 16,00 | 17,00 | 18,00 | 2,00 |
|  | 16,0 | 2 | 22,00 | 26,00 | 24,00 | 1,00 |
|  | 16,0 | 1 | 13,00 | 12,00 | 12,00 | 1,00 |
|  | 17,0 | 1 | 15,00 | 21,00 | 23,00 | 1,00 |
|  | 16,0 | 1 | 12,00 | 24,00 | 18,00 | 1,00 |
|  | 18,0 | 1 | 22,00 | 24,00 | 19,00 | 2,00 |
|  | 16,0 | 1 | 9,00 | 16,00 | 30,00 | 2,00 |
|  | 15,0 | 2 | 5,00 | 17,00 | 16,00 | 2,00 |
|  | 17,0 | 1 | 10,00 | 17,00 | 27,00 | 2,00 |
|  | 15,0 | 1 | 18,00 | 22,00 | 24,00 | 3,00 |
|  | 16,0 | 1 | 4,00 | 32,00 | 32,00 | 2,00 |
|  | 15,0 | 1 | 2,00 | 3,00 | ,00 | 1,00 |
|  | 16,0 | 1 | 14,00 | 11,00 | 7,00 | 1,00 |
|  | 15,0 | 1 | 12,00 | 15,00 | 22,00 | 2,00 |
|  | 15,0 | 1 | 12,00 | 15,00 | 23,00 | 2,00 |
|  | 15,0 | 1 | 7,00 | 4,00 | 25,00 | 3,00 |
|  | 16,0 | 2 | 12,00 | 8,00 | 15,00 | 1,00 |
|  | 15,0 | 2 | 23,00 | 22,00 | 28,00 | 1,00 |
|  | 15,0 | 1 | 12,00 | 16,00 | 15,00 | 1,00 |
|  | 17,0 | 1 | 22,00 | 9,00 | 7,00 | 1,00 |
|  | 16,0 | 1 | 14,00 | 29,00 | 24,00 | 1,00 |
|  | 16,0 | 1 | 20,00 | 25,00 | 32,00 | 1,00 |
|  | 15,0 | 1 | 16,00 | 28,00 | 22,00 | 2,00 |
|  | 15,0 | 1 | 9,00 | 12,00 | 17,00 | 1,00 |
|  | 16,0 | 2 | 5,00 | 3,00 | 25,00 | 1,00 |
|  | 16,0 | 1 | 19,00 | 13,00 | 14,00 | 1,00 |
|  | 15,0 | 1 | 17,00 | 12,00 | 10,00 | 1,00 |
|  | 16,0 | 1 | 12,00 | 15,00 | 21,00 | 1,00 |
|  | 16,0 | 1 | ,00 | 4,00 | 5,00 | 1,00 |
|  | 16,0 | 1 | 16,00 | 18,00 | 21,00 | 2,00 |
|  | 16,0 | 1 | 19,00 | 23,00 | 25,00 | 2,00 |
|  | 15,0 | 1 | 20,00 | 18,00 | 25,00 | 1,00 |
|  | 15,0 | 1 | 24,00 | 19,00 | 16,00 | 1,00 |
|  | 15,0 | 1 | 23,00 | 20,00 | 28,00 | 1,00 |
|  | 15,0 | 2 | 12,00 | 4,00 | 11,00 | 1,00 |
|  | 16,0 | 1 | 10,00 | 26,00 | 28,00 | 2,00 |
|  | 16,0 | 1 | ,00 | 16,00 | 32,00 | 1,00 |
|  | 17,0 | 1 | 24,00 | 27,00 | 12,00 | 1,00 |
|  | 15,0 | 1 | 8,00 | 26,00 | 24,00 | 2,00 |
|  | 17,0 | 1 | 1,00 | 16,00 | 10,00 | 1,00 |
|  | 15,0 | 1 | 20,00 | 28,00 | 20,00 | 1,00 |
|  | 15,0 | 1 | 21,00 | 28,00 | 30,00 | 1,00 |
|  | 17,0 | 1 | 13,00 | 16,00 | 27,00 | 2,00 |
|  | 15,0 | 1 | 19,00 | 23,00 | 27,00 | 1,00 |
|  | 16,0 | 1 | 15,00 | 17,00 | 22,00 | 1,00 |
|  | 15,0 | 1 | 3,00 | 16,00 | 15,00 | 1,00 |
|  | 17,0 | 2 | 13,00 | 16,00 | 20,00 | 3,00 |
|  | 15,0 | 1 | 7,00 | 22,00 | 15,00 | 1,00 |
|  | 16,0 | 1 | 10,00 | 13,00 | 14,00 | 1,00 |
|  | 16,0 | 1 | 12,00 | 32,00 | 28,00 | 1,00 |
|  | 15,0 | 1 | 14,00 | 17,00 | 9,00 | 1,00 |
|  | 15,0 | 2 | 20,00 | 16,00 | 21,00 | 1,00 |
|  | 17,0 | 1 | 10,00 | 16,00 | 17,00 | 2,00 |
|  | 15,0 | 1 | 8,00 | 15,00 | 24,00 | 1,00 |
|  | 16,0 | 1 | 13,00 | 15,00 | 22,00 | 1,00 |
|  | 15,0 | 1 | 13,00 | 18,00 | 24,00 | 1,00 |
|  | 16,0 | 1 | 20,00 | 17,00 | 24,00 | 1,00 |
|  | 17,0 | 1 | 10,00 | 15,00 | 16,00 | 1,00 |
|  | 15,0 | 1 | 15,00 | 26,00 | 27,00 | 1,00 |
|  | 16,0 | 1 | 20,00 | 22,00 | 28,00 | 1,00 |
|  | 15,0 | 1 | 7,00 | 22,00 | 25,00 | 1,00 |
|  | 16,0 | 1 | 18,00 | 18,00 | 12,00 | 1,00 |
|  | 18,0 | 1 | 19,00 | 22,00 | 23,00 | 1,00 |
|  | 15,0 | 1 | 26,00 | 22,00 | 28,00 | 1,00 |
|  | 16,0 | 1 | 15,00 | 22,00 | 22,00 | 1,00 |
|  | 15,0 | 1 | 13,00 | 11,00 | 10,00 | 1,00 |
|  | 15,0 | 1 | 13,00 | 19,00 | 19,00 | 1,00 |
|  | 17,0 | 1 | 25,00 | 25,00 | 21,00 | 1,00 |
|  | 15,0 | 1 | 22,00 | 21,00 | 22,00 | 1,00 |
|  | 15,0 | 1 | 23,00 | 20,00 | 22,00 | 2,00 |
|  | 15,0 | 1 | 10,00 | 5,00 | 10,00 | 2,00 |
|  | 16,0 | 1 | 15,00 | 19,00 | 17,00 | 1,00 |
|  | 17,0 | 1 | 8,00 | 10,00 | 20,00 | 2,00 |
|  | 15,0 | 1 | 3,00 | 17,00 | 28,00 | 3,00 |
|  | 15,0 | 1 | 9,00 | 17,00 | 23,00 | 1,00 |
|  | 16,0 | 1 | 15,00 | 20,00 | 18,00 | 1,00 |
|  | 16,0 | 1 | 18,00 | 19,00 | 26,00 | 1,00 |
|  | 16,0 | 1 | 12,00 | 18,00 | 17,00 | 1,00 |
|  | 16,0 | 1 | 16,00 | 12,00 | 22,00 | 1,00 |
|  | 15,0 | 1 | 7,00 | 29,00 | 30,00 | 1,00 |
|  | 16,0 | 1 | 12,00 | 13,00 | 31,00 | 2,00 |
|  | 16,0 | 2 | 29,00 | 25,00 | 31,00 | 2,00 |
|  | 16,0 | 1 | 16,00 | 21,00 | 28,00 | 1,00 |
|  | 16,0 | 1 | 14,00 | 17,00 | 26,00 | 3,00 |
|  | 15,0 | 1 | 7,00 | 11,00 | 29,00 | 1,00 |
|  | 16,0 | 1 | 11,00 | 14,00 | 24,00 | 3,00 |
|  | 15,0 | 1 | 11,00 | 19,00 | 25,00 | 1,00 |
|  | 16,0 | 1 | 8,00 | 16,00 | 24,00 | 2,00 |
|  | 14,0 | 1 | 10,00 | 9,00 | 13,00 | 1,00 |
|  | 18,0 | 1 | 20,00 | 22,00 | 22,00 | 2,00 |
|  | 15,0 | 1 | 10,00 | 14,00 | 10,00 | 1,00 |
|  | 16,0 | 2 | 10,00 | 24,00 | 26,00 | 1,00 |
|  | 16,0 | 1 | 11,00 | 14,00 | 17,00 | 3,00 |
|  | 16,0 | 1 | 8,00 | 15,00 | 25,00 | 1,00 |
|  | 16,0 | 1 | 8,00 | 14,00 | 22,00 | 1,00 |
|  | 17,0 | 1 | 15,00 | 15,00 | 24,00 | 1,00 |
|  | 16,0 | 1 | 16,00 | 14,00 | 25,00 | 1,00 |
|  | 16,0 | 1 | 16,00 | 12,00 | 20,00 | 2,00 |
|  | 16,0 | 1 | 13,00 | 14,00 | 16,00 | 1,00 |
|  | 16,0 | 1 | 12,00 | 24,00 | 24,00 | 1,00 |
|  | 16,0 | 1 | 20,00 | 19,00 | 15,00 | 3,00 |
|  | 17,0 | 1 | 5,00 | 9,00 | 28,00 | 2,00 |
|  | 16,0 | 1 | 15,00 | 29,00 | 30,00 | 1,00 |
|  | 16,0 | 1 | 6,00 | 8,00 | 10,00 | 2,00 |
|  | 18,0 | 1 | 3,00 | 19,00 | 12,00 | 1,00 |
|  | 16,0 | 1 | 15,00 | 24,00 | 29,00 | 1,00 |
|  | 16,0 | 1 | 16,00 | 9,00 | 26,00 | 1,00 |
|  | 16,0 | 1 | 12,00 | 12,00 | 19,00 | 1,00 |
|  | 16,0 | 1 | 14,00 | 20,00 | 20,00 | 1,00 |
|  | 16,0 | 1 | 9,00 | 11,00 | 28,00 | 1,00 |
|  | 16,0 | 1 | 27,00 | 13,00 | 9,00 | 1,00 |
|  | 16,0 | 1 | 20,00 | 23,00 | 30,00 | 1,00 |
|  | 17,0 | 1 | 21,00 | 21,00 | 21,00 | 1,00 |
|  | 16,0 | 1 | 32,00 | 22,00 | 25,00 | 2,00 |
|  | 17,0 | 1 | 12,00 | 13,00 | 20,00 | 1,00 |
|  | 17,0 | 1 | 10,00 | 20,00 | 27,00 | 3,00 |
|  | 16,0 | 1 | 11,00 | 17,00 | 25,00 | 1,00 |
|  | 18,0 | 1 | 7,00 | 15,00 | 18,00 | 1,00 |
|  | 17,0 | 1 | 11,00 | 20,00 | 25,00 | 2,00 |
|  | 16,0 | 1 | 7,00 | 17,00 | 20,00 | 1,00 |
|  | 17,0 | 1 | 3,00 | 18,00 | 25,00 | 1,00 |
|  | 16,0 | 1 | 6,00 | 10,00 | 20,00 | 2,00 |
|  | 16,0 | 1 | 22,00 | 16,00 | 20,00 | 1,00 |
|  | 16,0 | 1 | 16,00 | 11,00 | 19,00 | 1,00 |
|  | 16,0 | 1 | 2,00 | 19,00 | 12,00 | 3,00 |
|  | 16,0 | 1 | 11,00 | 13,00 | 15,00 | 1,00 |
|  | 17,0 | 1 | 19,00 | 16,00 | 29,00 | 2,00 |
|  | 16,0 | 1 | 13,00 | 19,00 | 14,00 | 3,00 |
|  | 16,0 | 1 | 7,00 | 17,00 | 27,00 | 3,00 |
|  | 16,0 | 1 | 1,00 | 12,00 | 4,00 | 1,00 |
|  | 16,0 | 1 | 11,00 | 12,00 | 15,00 | 1,00 |
|  | 16,0 | 1 | 1,00 | 8,00 | 11,00 | 3,00 |
|  | 16,0 | 1 | 10,00 | 20,00 | 22,00 | 2,00 |
|  | 16,0 | 1 | 10,00 | 11,00 | 12,00 | 3,00 |
|  | 16,0 | 1 | 15,00 | 17,00 | 11,00 | 1,00 |
|  | 16,0 | 1 | 11,00 | 13,00 | 19,00 | 1,00 |
|  | 16,0 | 1 | 14,00 | 22,00 | 15,00 | 2,00 |
|  | 16,0 | 1 | 12,00 | 4,00 | 10,00 | 1,00 |
|  | 16,0 | 1 | 9,00 | 12,00 | 12,00 | 1,00 |
|  | 18,0 | 1 | 19,00 | 20,00 | 21,00 | 1,00 |
|  | 16,0 | 1 | 13,00 | 15,00 | 16,00 | 2,00 |
|  | 17,0 | 1 | 23,00 | 16,00 | 24,00 | 1,00 |
|  | 16,0 | 1 | 4,00 | 8,00 | 5,00 | 3,00 |
|  | 16,0 | 1 | 23,00 | 3,00 | 23,00 | 2,00 |
|  | 16,0 | 1 | 15,00 | 11,00 | 22,00 | 2,00 |
|  | 17,0 | 1 | 25,00 | 24,00 | 22,00 | 1,00 |
|  | 16,0 | 1 | 12,00 | 14,00 | 19,00 | 1,00 |
|  | 16,0 | 1 | 27,00 | 17,00 | 15,00 | 2,00 |
|  | 16,0 | 1 | 16,00 | 18,00 | 18,00 | 2,00 |
|  | 16,0 | 1 | 18,00 | 17,00 | 15,00 | 1,00 |
|  | 16,0 | 1 | 6,00 | 8,00 | 24,00 | 1,00 |
|  | 16,0 | 1 | 19,00 | 30,00 | 31,00 | 1,00 |
|  | 16,0 | 1 | 16,00 | 26,00 | 21,00 | 1,00 |
|  | 18,0 | 1 | 12,00 | 10,00 | 18,00 | 3,00 |
|  | 17,0 | 1 | 20,00 | 13,00 | 12,00 | 1,00 |
|  | 18,0 | 1 | 11,00 | 13,00 | 17,00 | 1,00 |
|  | 18,0 | 1 | 14,00 | 18,00 | 17,00 | 1,00 |
|  | 17,0 | 1 | 7,00 | 8,00 | 17,00 | 2,00 |
|  | 17,0 | 1 | 21,00 | 18,00 | 29,00 | 1,00 |
|  | 17,0 | 1 | 9,00 | 21,00 | 21,00 | 2,00 |
|  | 16,0 | 1 | 6,00 | 18,00 | 19,00 | 3,00 |
|  | 16,0 | 1 | 23,00 | 21,00 | 22,00 | 1,00 |
|  | 17,0 | 1 | 14,00 | 17,00 | 20,00 | 3,00 |
|  | 16,0 | 1 | 6,00 | 20,00 | 20,00 | 1,00 |
|  | 16,0 | 1 | 5,00 | 22,00 | 19,00 | 1,00 |
|  | 16,0 | 1 | 10,00 | 19,00 | 15,00 | 1,00 |
|  | 17,0 | 1 | 14,00 | 10,00 | 30,00 | 1,00 |
|  | 16,0 | 1 | 20,00 | 15,00 | 19,00 | 1,00 |
|  | 16,0 | 1 | 19,00 | 18,00 | 23,00 | 1,00 |
|  | 16,0 | 1 | 23,00 | 21,00 | 21,00 | 1,00 |
|  | 17,0 | 1 | 10,00 | 17,00 | 18,00 | 1,00 |
|  | 16,0 | 1 | 15,00 | 14,00 | 4,00 | 1,00 |
|  | 17,0 | 1 | 17,00 | 22,00 | 28,00 | 1,00 |
|  | 17,0 | 1 | 8,00 | 17,00 | 23,00 | 1,00 |
|  | 18,0 | 1 | 16,00 | 29,00 | 26,00 | 2,00 |
|  | 17,0 | 1 | 18,00 | 23,00 | 20,00 | 1,00 |
|  | 17,0 | 1 | 15,00 | 16,00 | 18,00 | 1,00 |
|  | 18,0 | 1 | 18,00 | 3,00 | 11,00 | 1,00 |
|  | 18,0 | 1 | 7,00 | 24,00 | 26,00 | 2,00 |
|  | 18,0 | 1 | 9,00 | 15,00 | 20,00 | 2,00 |
|  | 18,0 | 1 | 16,00 | 23,00 | 25,00 | 1,00 |
|  | 18,0 | 1 | 22,00 | 14,00 | 8,00 | 1,00 |
|  | 18,0 | 1 | 14,00 | 19,00 | 24,00 | 1,00 |
|  | 17,0 | 1 | 15,00 | 14,00 | 25,00 | 1,00 |
|  | 17,0 | 1 | 25,00 | 25,00 | 32,00 | 2,00 |
|  | 18,0 | 1 | 19,00 | 18,00 | 26,00 | 1,00 |
|  | 17,0 | 1 | 17,00 | 12,00 | 11,00 | 1,00 |
|  | 18,0 | 2 | 12,00 | 18,00 | 23,00 | 1,00 |
|  | 17,0 | 1 | 11,00 | 16,00 | 20,00 | 2,00 |
|  | 18,0 | 1 | 13,00 | 13,00 | 28,00 | 2,00 |
|  | 18,0 | 1 | 12,00 | 11,00 | 13,00 | 1,00 |
|  | 18,0 | 1 | 10,00 | 12,00 | 14,00 | 1,00 |
|  | 17,0 | 1 | 15,00 | 17,00 | 17,00 | 3,00 |
|  | 17,0 | 1 | 20,00 | 13,00 | 22,00 | 3,00 |
|  | 17,0 | 1 | 19,00 | 18,00 | 26,00 | 1,00 |
|  | 18,0 | 1 | 14,00 | 17,00 | 23,00 | 2,00 |
|  | 18,0 | 1 | 10,00 | 16,00 | 23,00 | 3,00 |
|  | 18,0 | 1 | 7,00 | 15,00 | 11,00 | 1,00 |
|  | 18,0 | 1 | 18,00 | 26,00 | 25,00 | 1,00 |
|  | 17,0 | 1 | 22,00 | 13,00 | 12,00 | 1,00 |
|  | 17,0 | 1 | 20,00 | 22,00 | 25,00 | 1,00 |
|  | 18,0 | 1 | 8,00 | 27,00 | 26,00 | 3,00 |
|  | 18,0 | 1 | 8,00 | 16,00 | 22,00 | 1,00 |
|  | 18,0 | 1 | ,00 | 20,00 | 30,00 | 1,00 |
|  | 18,0 | 1 | 11,00 | 14,00 | 21,00 | 1,00 |
|  | 17,0 | 1 | 16,00 | 22,00 | 17,00 | 1,00 |
|  | 18,0 | 1 | 13,00 | 11,00 | 22,00 | 1,00 |
|  | 18,0 | 1 | 9,00 | 16,00 | 20,00 | 1,00 |
|  | 17,0 | 1 | 13,00 | 20,00 | 16,00 | 3,00 |
|  | 18,0 | 1 | 14,00 | 12,00 | 23,00 | 1,00 |
|  | 17,0 | 1 | 15,00 | 19,00 | 14,00 | 1,00 |
|  | 18,0 | 1 | 19,00 | 15,00 | 20,00 | 2,00 |
|  | 17,0 | 1 | 19,00 | 13,00 | 19,00 | 2,00 |
|  | 18,0 | 1 | 20,00 | 19,00 | 20,00 | 1,00 |
|  | 18,0 | 1 | 9,00 | 17,00 | 12,00 | 1,00 |
|  | 18,0 | 1 | 16,00 | 12,00 | 18,00 | 1,00 |
|  | 18,0 | 1 | 10,00 | 19,00 | 20,00 | 1,00 |
|  | 18,0 | 1 | 7,00 | 22,00 | 22,00 | 2,00 |
|  | 17,0 | 1 | 15,00 | 16,00 | 22,00 | 1,00 |
|  | 17,0 | 1 | 20,00 | 19,00 | 28,00 | 1,00 |
|  | 18,0 | 1 | 23,00 | 15,00 | 15,00 | 1,00 |
|  | 18,0 | 1 | 20,00 | 22,00 | 29,00 | 1,00 |
|  | 17,0 | 1 | 12,00 | 6,00 | 17,00 | 1,00 |
|  | 17,0 | 1 | 14,00 | 11,00 | 21,00 | 2,00 |
|  | 17,0 | 1 | 5,00 | 26,00 | 28,00 | 1,00 |
|  | 18,0 | 1 | 16,00 | 25,00 | 10,00 | 1,00 |
|  | 17,0 | 1 | 18,00 | 16,00 | 19,00 | 2,00 |
|  | 17,0 | 1 | 7,00 | 14,00 | 20,00 | 1,00 |
|  | 16,0 | 1 | 17,00 | 11,00 | 16,00 | 1,00 |
|  | 17,0 | 1 | 27,00 | 18,00 | 22,00 | 1,00 |
|  | 17,0 | 1 | 13,00 | 21,00 | 26,00 | 3,00 |
|  | 18,0 | 1 | 17,00 | 19,00 | 18,00 | 1,00 |
|  | 17,0 | 1 | 7,00 | 17,00 | 21,00 | 1,00 |
|  | 17,0 | 1 | ,00 | 16,00 | 22,00 | 1,00 |
|  | 17,0 | 1 | 8,00 | 8,00 | 21,00 | 1,00 |
|  | 17,0 | 1 | 9,00 | 14,00 | 22,00 | 1,00 |
|  | 18,0 | 1 | 19,00 | 16,00 | 27,00 | 1,00 |
|  | 18,0 | 1 | 10,00 | 10,00 | 18,00 | 1,00 |
|  | 17,0 | 1 | ,00 | 5,00 | 23,00 | 1,00 |
|  | 17,0 | 1 | 8,00 | 19,00 | 24,00 | 1,00 |
|  | 18,0 | 1 | 14,00 | 18,00 | 25,00 | 2,00 |
|  | 17,0 | 1 | 14,00 | 16,00 | 26,00 | 2,00 |
|  | 17,0 | 1 | 26,00 | 28,00 | 28,00 | 1,00 |
|  | 17,0 | 1 | 22,00 | 19,00 | 32,00 | 1,00 |
|  | 17,0 | 1 | 15,00 | 13,00 | 18,00 | 2,00 |
|  | 18,0 | 1 | 1,00 | 22,00 | 12,00 | 1,00 |
|  | 17,0 | 1 | 18,00 | 24,00 | 28,00 | 2,00 |
|  | 17,0 | 1 | 14,00 | 23,00 | 24,00 | 2,00 |
|  | 17,0 | 1 | 16,00 | 20,00 | 17,00 | 3,00 |
|  | 17,0 | 1 | 12,00 | 26,00 | 25,00 | 1,00 |
|  | 17,0 | 1 | 15,00 | 14,00 | 23,00 | 1,00 |
|  | 18,0 | 1 | 7,00 | 10,00 | 18,00 | 1,00 |
|  | 18,0 | 1 | 13,00 | 18,00 | 27,00 | 2,00 |
|  | 16,0 | 1 | 6,00 | 14,00 | 20,00 | 1,00 |
|  | 17,0 | 1 | 19,00 | 16,00 | 27,00 | 1,00 |
|  | 18,0 | 1 | 19,00 | 24,00 | 22,00 | 2,00 |
|  | 18,0 | 1 | 19,00 | 32,00 | 26,00 | 1,00 |
|  | 17,0 | 1 | 17,00 | 13,00 | 22,00 | 2,00 |
|  | 18,0 | 1 | 25,00 | 15,00 | 20,00 | 2,00 |
|  | 17,0 | 1 | 9,00 | 17,00 | 26,00 | 1,00 |
|  | 17,0 | 1 | 15,00 | 9,00 | 13,00 | 1,00 |
|  | 18,0 | 1 | 19,00 | 23,00 | 28,00 | 1,00 |
|  | 18,0 | 1 | 14,00 | 23,00 | 25,00 | 1,00 |
|  | 17,0 | 1 | 12,00 | 16,00 | 23,00 | 1,00 |
|  | 18,0 | 1 | 24,00 | 28,00 | 26,00 | 2,00 |
|  | 17,0 | 1 | 23,00 | 20,00 | 28,00 | 3,00 |
|  | 17,0 | 1 | 14,00 | 22,00 | 14,00 | 1,00 |
|  | 17,0 | 1 | 15,00 | 20,00 | 25,00 | 2,00 |
|  | 17,0 | 1 | 11,00 | 11,00 | 23,00 | 1,00 |
|  | 18,0 | 1 | 8,00 | 23,00 | 24,00 | 1,00 |
|  | 17,0 | 1 | 15,00 | 3,00 | 27,00 | 1,00 |
|  | 17,0 | 1 | 6,00 | 17,00 | 25,00 | 1,00 |
|  | 17,0 | 1 | 16,00 | 12,00 | 22,00 | 1,00 |
|  | 18,0 | 1 | 25,00 | 25,00 | 27,00 | 2,00 |
|  | 17,0 | 1 | 14,00 | 27,00 | 31,00 | 2,00 |
|  | 18,0 | 1 | 23,00 | 17,00 | 26,00 | 1,00 |
|  | 18,0 | 1 | 8,00 | 12,00 | 14,00 | 1,00 |
|  | 18,0 | 1 | 15,00 | 21,00 | 19,00 | 1,00 |
|  | 17,0 | 1 | 22,00 | 21,00 | 12,00 | 1,00 |
|  | 18,0 | 1 | 22,00 | 27,00 | 27,00 | 1,00 |
|  | 18,0 | 1 | 32,00 | 17,00 | 14,00 | 1,00 |
|  | 17,0 | 1 | 11,00 | 8,00 | 9,00 | 2,00 |
|  | 18,0 | 1 | 18,00 | 21,00 | 29,00 | 1,00 |
|  | 18,0 | 1 | 16,00 | 10,00 | 23,00 | 1,00 |
|  | 18,0 | 1 | 11,00 | 17,00 | 20,00 | 1,00 |
|  | 18,0 | 1 | 1,00 | 18,00 | 21,00 | 2,00 |
|  | 18,0 | 1 | 20,00 | 16,00 | 18,00 | 1,00 |
|  | 18,0 | 1 | 10,00 | 19,00 | 32,00 | 1,00 |
|  | 17,0 | 1 | 7,00 | 18,00 | 24,00 | 1,00 |
|  | 18,0 | 1 | 19,00 | 20,00 | 16,00 | 1,00 |
|  | 18,0 | 1 | 9,00 | 20,00 | 27,00 | 1,00 |
|  | 17,0 | 1 | 19,00 | 29,00 | 28,00 | 1,00 |
|  | 17,0 | 1 | 26,00 | 8,00 | 17,00 | 1,00 |
|  | 17,0 | 1 | 9,00 | 16,00 | 15,00 | 1,00 |
|  | 18,0 | 1 | 32,00 | 10,00 | 28,00 | 2,00 |
|  | 18,0 | 1 | 7,00 | 15,00 | 18,00 | 1,00 |
|  | 17,0 | 1 | 13,00 | 21,00 | 19,00 | 1,00 |
|  | 18,0 | 1 | 19,00 | 13,00 | 19,00 | 1,00 |
|  | 18,0 | 1 | 12,00 | 18,00 | 17,00 | 1,00 |
|  | 18,0 | 1 | 15,00 | 11,00 | 18,00 | 2,00 |
|  | 18,0 | 1 | 6,00 | 16,00 | 25,00 | 1,00 |
|  | 17,0 | 1 | 13,00 | 15,00 | 18,00 | 1,00 |
|  | 18,0 | 1 | 14,00 | 18,00 | 23,00 | 3,00 |
|  | 17,0 | 1 | 14,00 | 25,00 | 23,00 | 1,00 |
|  | 17,0 | 1 | 24,00 | 12,00 | 23,00 | 1,00 |
|  | 18,0 | 1 | 10,00 | 24,00 | 28,00 | 1,00 |
|  | 18,0 | 1 | 20,00 | 22,00 | 28,00 | 1,00 |
|  | 17,0 | 1 | 10,00 | 19,00 | 24,00 | 1,00 |
|  | 18,0 | 1 | 18,00 | 22,00 | 18,00 | 2,00 |
|  | 18,0 | 1 | 11,00 | 19,00 | 17,00 | 1,00 |
|  | 17,0 | 1 | 15,00 | 19,00 | 21,00 | 1,00 |
|  | 17,0 | 1 | 14,00 | 21,00 | 21,00 | 1,00 |
|  | 17,0 | 1 | 20,00 | 30,00 | 32,00 | 1,00 |
|  | 18,0 | 1 | 28,00 | 18,00 | 27,00 | 2,00 |
|  | 17,0 | 1 | 22,00 | 24,00 | 28,00 | 2,00 |
|  | 17,0 | 1 | 9,00 | 12,00 | 19,00 | 1,00 |
|  | 18,0 | 1 | 22,00 | 24,00 | 29,00 | 1,00 |
|  | 13,0 | 1 | 8,00 | 12,00 | 19,00 | 1,00 |
|  | 12,0 | 1 | 22,00 | 26,00 | 21,00 | 2,00 |
|  | 12,0 | 2 | 12,00 | 16,00 | 15,00 | 1,00 |
|  | 12,0 | 1 | 16,00 | 19,00 | 29,00 | 3,00 |
|  | 12,0 | 2 | 11,00 | 19,00 | 27,00 | 1,00 |
|  | 12,0 | 1 | 2,00 | 9,00 | 16,00 | 1,00 |
|  | 12,0 | 2 | 6,00 | 23,00 | 10,00 | 2,00 |
|  | 12,0 | 2 | 22,00 | 28,00 | 32,00 | 3,00 |
|  | 12,0 | 2 | 20,00 | 20,00 | 19,00 | 2,00 |
|  | 13,0 | 1 | 25,00 | 26,00 | 10,00 | 1,00 |
|  | 12,0 | 2 | 18,00 | 19,00 | 27,00 | 1,00 |
|  | 13,0 | 1 | 22,00 | 13,00 | 28,00 | 1,00 |
|  | 13,0 | 1 | 19,00 | 15,00 | 17,00 | 1,00 |
|  | 12,0 | 1 | 15,00 | 17,00 | 25,00 | 1,00 |
|  | 12,0 | 2 | 14,00 | 26,00 | 25,00 | 1,00 |
|  | 12,0 | 1 | 14,00 | 14,00 | 12,00 | 1,00 |
|  | 12,0 | 1 | 32,00 | 28,00 | 31,00 | 1,00 |
|  | 13,0 | 2 | 18,00 | 11,00 | 15,00 | 1,00 |
|  | 12,0 | 2 | ,00 | 7,00 | 13,00 | 2,00 |
|  | 12,0 | 1 | 8,00 | 11,00 | 14,00 | 3,00 |
|  | 12,0 | 1 | 21,00 | 20,00 | 22,00 | 1,00 |
|  | 12,0 | 2 | 17,00 | 14,00 | 26,00 | 1,00 |
|  | 13,0 | 2 | 22,00 | 14,00 | 28,00 | 3,00 |
|  | 13,0 | 1 | 12,00 | 14,00 | 15,00 | 1,00 |
|  | 13,0 | 1 | 13,00 | 26,00 | 17,00 | 1,00 |
|  | 12,0 | 2 | 24,00 | 15,00 | 23,00 | 1,00 |
|  | 13,0 | 1 | 5,00 | 15,00 | 12,00 | 3,00 |
|  | 13,0 | 2 | 9,00 | 18,00 | 24,00 | 1,00 |
|  | 12,0 | 1 | 17,00 | 7,00 | 8,00 | 2,00 |
|  | 12,0 | 2 | 23,00 | 20,00 | 22,00 | 1,00 |
|  | 13,0 | 2 | 12,00 | 12,00 | 18,00 | 1,00 |
|  | 13,0 | 1 | 12,00 | 18,00 | 19,00 | 1,00 |
|  | 12,0 | 1 | 8,00 | 4,00 | 6,00 | 1,00 |
|  | 12,0 | 1 | 8,00 | 18,00 | 18,00 | 1,00 |
|  | 12,0 | 1 | 21,00 | 13,00 | 15,00 | 2,00 |
|  | 13,0 | 1 | 5,00 | 19,00 | 21,00 | 3,00 |
|  | 12,0 | 1 | 13,00 | 17,00 | 16,00 | 2,00 |
|  | 12,0 | 2 | ,00 | 2,00 | 7,00 | 2,00 |
|  | 12,0 | 2 | 12,00 | 24,00 | 24,00 | 3,00 |
|  | 12,0 | 1 | ,00 | ,00 | ,00 | 1,00 |
|  | 12,0 | 1 | 12,00 | 14,00 | 14,00 | 3,00 |
|  | 12,0 | 1 | 2,00 | 8,00 | 19,00 | 1,00 |
|  | 12,0 | 2 | 15,00 | 17,00 | 16,00 | 1,00 |
|  | 12,0 | 2 | 14,00 | 20,00 | 30,00 | 2,00 |
|  | 12,0 | 1 | 25,00 | 25,00 | 22,00 | 2,00 |
|  | 12,0 | 2 | 21,00 | 18,00 | 27,00 | 3,00 |
|  | 12,0 | 2 | 19,00 | 22,00 | 27,00 | 2,00 |
|  | 12,0 | 1 | 13,00 | 21,00 | 17,00 | 1,00 |
|  | 12,0 | 2 | 8,00 | 13,00 | 20,00 | 1,00 |
|  | 12,0 | 1 | 6,00 | 13,00 | 18,00 | 3,00 |
|  | 12,0 | 2 | 21,00 | 29,00 | 27,00 | 1,00 |
|  | 12,0 | 1 | 15,00 | 23,00 | 17,00 | 1,00 |
|  | 12,0 | 2 | 19,00 | 8,00 | 13,00 | 2,00 |
|  | 12,0 | 2 | 9,00 | 21,00 | 24,00 | 3,00 |
|  | 12,0 | 2 | 13,00 | 23,00 | 22,00 | 1,00 |
|  | 12,0 | 1 | 20,00 | 27,00 | 23,00 | 1,00 |
|  | 12,0 | 1 | 13,00 | 16,00 | 23,00 | 2,00 |
|  | 12,0 | 2 | 28,00 | 19,00 | 27,00 | 1,00 |
|  | 13,0 | 2 | 21,00 | 16,00 | 23,00 | 1,00 |
|  | 13,0 | 1 | 11,00 | 23,00 | 28,00 | 1,00 |
|  | 12,0 | 2 | 16,00 | 14,00 | 25,00 | 1,00 |
|  | 12,0 | 1 | 22,00 | 23,00 | 22,00 | 1,00 |
|  | 12,0 | 2 | 16,00 | 13,00 | 19,00 | 1,00 |
|  | 12,0 | 1 | 11,00 | 20,00 | 15,00 | 1,00 |
|  | 12,0 | 1 | 19,00 | 21,00 | 20,00 | 2,00 |
|  | 12,0 | 2 | 6,00 | 24,00 | 24,00 | 1,00 |
|  | 14,0 | 2 | 27,00 | 23,00 | 24,00 | 1,00 |
|  | 13,0 | 1 | 10,00 | 12,00 | 10,00 | 1,00 |
|  | 13,0 | 2 | 25,00 | 32,00 | 32,00 | 3,00 |
|  | 13,0 | 1 | 9,00 | 27,00 | 23,00 | 3,00 |
|  | 13,0 | 2 | 13,00 | 18,00 | 21,00 | 3,00 |
|  | 13,0 | 2 | 29,00 | 26,00 | 22,00 | 3,00 |
|  | 14,0 | 1 | 21,00 | 17,00 | 29,00 | 2,00 |
|  | 13,0 | 2 | 11,00 | 7,00 | 27,00 | 1,00 |
|  | 13,0 | 1 | 12,00 | 19,00 | 24,00 | 1,00 |
|  | 14,0 | 1 | 9,00 | 14,00 | 26,00 | 3,00 |
|  | 13,0 | 2 | 25,00 | 20,00 | 26,00 | 1,00 |
|  | 14,0 | 2 | 13,00 | 9,00 | 4,00 | 1,00 |
|  | 13,0 | 2 | 13,00 | 17,00 | 17,00 | 1,00 |
|  | 14,0 | 2 | 20,00 | 7,00 | 13,00 | 1,00 |
|  | 13,0 | 2 | 12,00 | 13,00 | 28,00 | 1,00 |
|  | 13,0 | 2 | 25,00 | 22,00 | 26,00 | 1,00 |
|  | 13,0 | 1 | 8,00 | 19,00 | 25,00 | 1,00 |
|  | 13,0 | 1 | 7,00 | 17,00 | 25,00 | 2,00 |
|  | 13,0 | 2 | 17,00 | 16,00 | 28,00 | 1,00 |
|  | 13,0 | 1 | 12,00 | 28,00 | 21,00 | 1,00 |
|  | 13,0 | 1 | 26,00 | 20,00 | 26,00 | 1,00 |
|  | 14,0 | 1 | 25,00 | 14,00 | 20,00 | 1,00 |
|  | 13,0 | 2 | 17,00 | 14,00 | 10,00 | 1,00 |
|  | 14,0 | 1 | 7,00 | 20,00 | 26,00 | 1,00 |
|  | 14,0 | 1 | 17,00 | 24,00 | 25,00 | 1,00 |
|  | 13,0 | 2 | 24,00 | 15,00 | 23,00 | 3,00 |
|  | 13,0 | 1 | 17,00 | 20,00 | 24,00 | 1,00 |
|  | 14,0 | 1 | 12,00 | 10,00 | 13,00 | 1,00 |
|  | 14,0 | 1 | 25,00 | 22,00 | 31,00 | 1,00 |
|  | 14,0 | 2 | 14,00 | 10,00 | 12,00 | 2,00 |
|  | 13,0 | 2 | 16,00 | 22,00 | 18,00 | 2,00 |
|  | 13,0 | 2 | 15,00 | 19,00 | 21,00 | 3,00 |
|  | 13,0 | 1 | 7,00 | 10,00 | 30,00 | 3,00 |
|  | 13,0 | 2 | 23,00 | 23,00 | 14,00 | 1,00 |
|  | 14,0 | 1 | 11,00 | 18,00 | 17,00 | 1,00 |
|  | 14,0 | 1 | 18,00 | 17,00 | 20,00 | 1,00 |
|  | 13,0 | 1 | 19,00 | 25,00 | 27,00 | 1,00 |
|  | 13,0 | 2 | 9,00 | 20,00 | 23,00 | 2,00 |
|  | 14,0 | 1 | 30,00 | 23,00 | 27,00 | 2,00 |
|  | 13,0 | 2 | 15,00 | 5,00 | 22,00 | 3,00 |
|  | 13,0 | 1 | 22,00 | 20,00 | 22,00 | 1,00 |
|  | 13,0 | 2 | 15,00 | 15,00 | 21,00 | 1,00 |
|  | 13,0 | 2 | 11,00 | 18,00 | 19,00 | 1,00 |
|  | 13,0 | 2 | 10,00 | 8,00 | 18,00 | 1,00 |
|  | 13,0 | 2 | 10,00 | 12,00 | 20,00 | 1,00 |
|  | 14,0 | 2 | 22,00 | 21,00 | 26,00 | 1,00 |
|  | 14,0 | 1 | 27,00 | 22,00 | 27,00 | 1,00 |
|  | 13,0 | 1 | 17,00 | 17,00 | 20,00 | 1,00 |
|  | 13,0 | 1 | 11,00 | 15,00 | 11,00 | 1,00 |
|  | 13,0 | 1 | 25,00 | 23,00 | 26,00 | 1,00 |
|  | 13,0 | 1 | 18,00 | 17,00 | 22,00 | 1,00 |
|  | 13,0 | 1 | 10,00 | 17,00 | 28,00 | 2,00 |
|  | 13,0 | 2 | 12,00 | 15,00 | 25,00 | 3,00 |
|  | 13,0 | 2 | 18,00 | 18,00 | 29,00 | 3,00 |
|  | 13,0 | 1 | 5,00 | 11,00 | 29,00 | 1,00 |
|  | 13,0 | 1 | 10,00 | 10,00 | 14,00 | 1,00 |
|  | 13,0 | 2 | 22,00 | 19,00 | 29,00 | 1,00 |
|  | 14,0 | 2 | 19,00 | 23,00 | 21,00 | 1,00 |
|  | 13,0 | 2 | 15,00 | 18,00 | 17,00 | 1,00 |
|  | 13,0 | 2 | 17,00 | 15,00 | 23,00 | 1,00 |
|  | 14,0 | 2 | 18,00 | 14,00 | 14,00 | 1,00 |
|  | 14,0 | 2 | 25,00 | 6,00 | 27,00 | 1,00 |
|  | 14,0 | 2 | 15,00 | 20,00 | 27,00 | 1,00 |
|  | 14,0 | 2 | 11,00 | 13,00 | 22,00 | 1,00 |
|  | 15,0 | 1 | 4,00 | 8,00 | 16,00 | 3,00 |
|  | 15,0 | 2 | 15,00 | 9,00 | 20,00 | 3,00 |
|  | 14,0 | 2 | 15,00 | 11,00 | 18,00 | 1,00 |
|  | 14,0 | 2 | 20,00 | 24,00 | 25,00 | 2,00 |
|  | 14,0 | 1 | 19,00 | 24,00 | 20,00 | 1,00 |
|  | 14,0 | 1 | 18,00 | 27,00 | 30,00 | 1,00 |
|  | 15,0 | 2 | 11,00 | 5,00 | 18,00 | 1,00 |
|  | 15,0 | 1 | 5,00 | 8,00 | 8,00 | 1,00 |
|  | 14,0 | 2 | 29,00 | 21,00 | 32,00 | 2,00 |
|  | 14,0 | 1 | 19,00 | 5,00 | 25,00 | 2,00 |
|  | 14,0 | 2 | 16,00 | 19,00 | 30,00 | 1,00 |
|  | 14,0 | 2 | 15,00 | 13,00 | 14,00 | 1,00 |
|  | 14,0 | 2 | 22,00 | 22,00 | 21,00 | 2,00 |
|  | 14,0 | 1 | 8,00 | 20,00 | 16,00 | 1,00 |
|  | 15,0 | 1 | 5,00 | 4,00 | 12,00 | 1,00 |
|  | 14,0 | 1 | 3,00 | 16,00 | 27,00 | 2,00 |
|  | 15,0 | 2 | 12,00 | 16,00 | 25,00 | 1,00 |
|  | 14,0 | 2 | 4,00 | 12,00 | 23,00 | 3,00 |
|  | 14,0 | 1 | 17,00 | 13,00 | 18,00 | 1,00 |
|  | 15,0 | 2 | 16,00 | 25,00 | 30,00 | 2,00 |
|  | 14,0 | 2 | 22,00 | 20,00 | 29,00 | 2,00 |
|  | 14,0 | 2 | 13,00 | 11,00 | 25,00 | 1,00 |
|  | 15,0 | 1 | 3,00 | 15,00 | 20,00 | 1,00 |
|  | 14,0 | 2 | 14,00 | 14,00 | 17,00 | 1,00 |
|  | 14,0 | 1 | 11,00 | 16,00 | 21,00 | 3,00 |
|  | 14,0 | 2 | 26,00 | 18,00 | 21,00 | 1,00 |
|  | 14,0 | 1 | 18,00 | 25,00 | 28,00 | 1,00 |
|  | 14,0 | 1 | 17,00 | 18,00 | 29,00 | 1,00 |
|  | 14,0 | 1 | 13,00 | 13,00 | 18,00 | 1,00 |
|  | 14,0 | 2 | 17,00 | 21,00 | 24,00 | 1,00 |
|  | 14,0 | 2 | 11,00 | 16,00 | 26,00 | 1,00 |
|  | 14,0 | 2 | 18,00 | 14,00 | 19,00 | 1,00 |
|  | 15,0 | 2 | 3,00 | 4,00 | ,00 | 1,00 |
|  | 15,0 | 1 | 16,00 | 13,00 | 20,00 | 1,00 |
|  | 15,0 | 1 | 21,00 | 24,00 | 21,00 | 2,00 |
|  | 14,0 | 2 | 10,00 | 20,00 | 31,00 | 1,00 |
|  | 15,0 | 1 | 13,00 | 13,00 | 22,00 | 2,00 |
|  | 14,0 | 2 | 21,00 | 4,00 | 7,00 | 1,00 |
|  | 15,0 | 2 | 15,00 | 17,00 | 20,00 | 1,00 |
|  | 14,0 | 2 | 13,00 | 22,00 | 21,00 | 1,00 |
|  | 14,0 | 1 | 14,00 | 1,00 | 1,00 | 2,00 |
|  | 14,0 | 1 | 9,00 | 18,00 | 28,00 | 1,00 |
|  | 14,0 | 1 | 7,00 | 17,00 | 22,00 | 1,00 |
|  | 14,0 | 1 | 31,00 | 10,00 | 12,00 | 1,00 |
|  | 14,0 | 2 | 21,00 | 23,00 | 29,00 | 1,00 |
|  | 14,0 | 1 | 9,00 | 17,00 | 24,00 | 2,00 |
|  | 14,0 | 2 | 24,00 | 16,00 | 20,00 | 1,00 |
|  | 14,0 | 2 | 25,00 | 24,00 | 27,00 | 2,00 |
|  | 14,0 | 1 | 12,00 | 10,00 | 21,00 | 1,00 |
|  | 14,0 | 1 | 15,00 | 11,00 | 16,00 | 2,00 |
|  | 15,0 | 1 | 10,00 | 24,00 | 27,00 | 1,00 |
|  | 14,0 | 2 | 24,00 | 18,00 | 29,00 | 1,00 |
|  | 15,0 | 1 | 19,00 | 26,00 | 27,00 | 3,00 |
|  | 17,0 | 2 | 26,00 | 11,00 | 17,00 | 1,00 |
|  | 15,0 | 1 | 10,00 | 19,00 | 16,00 | 3,00 |
|  | 15,0 | 1 | 18,00 | 19,00 | 26,00 | 2,00 |
|  | 15,0 | 2 | 24,00 | 10,00 | 20,00 | 1,00 |
|  | 15,0 | 1 | 16,00 | 18,00 | 25,00 | 1,00 |
|  | 16,0 | 2 | 13,00 | 10,00 | 26,00 | 1,00 |
|  | 15,0 | 2 | 14,00 | 1,00 | 1,00 | 1,00 |
|  | 15,0 | 2 | 25,00 | 8,00 | 26,00 | 1,00 |
|  | 15,0 | 1 | 13,00 | 7,00 | 8,00 | 1,00 |
|  | 15,0 | 1 | 22,00 | 15,00 | 26,00 | 1,00 |
|  | 15,0 | 1 | 5,00 | 6,00 | 4,00 | 1,00 |
|  | 16,0 | 2 | 10,00 | 14,00 | 21,00 | 1,00 |
|  | 16,0 | 2 | 24,00 | 16,00 | 25,00 | 1,00 |
|  | 15,0 | 2 | 7,00 | 22,00 | 30,00 | 1,00 |
|  | 15,0 | 2 | 8,00 | 24,00 | 26,00 | 1,00 |
|  | 15,0 | 1 | 16,00 | 25,00 | 27,00 | 1,00 |
|  | 16,0 | 2 | 16,00 | 22,00 | 17,00 | 1,00 |
|  | 15,0 | 2 | 20,00 | 17,00 | 23,00 | 1,00 |
|  | 16,0 | 2 | 15,00 | 16,00 | 28,00 | 1,00 |
|  | 15,0 | 1 | 20,00 | 21,00 | 29,00 | 2,00 |
|  | 16,0 | 1 | ,00 | 3,00 | 4,00 | 1,00 |
|  | 15,0 | 2 | 18,00 | 16,00 | 13,00 | 1,00 |
|  | 15,0 | 2 | 11,00 | 16,00 | 19,00 | 1,00 |
|  | 15,0 | 2 | 26,00 | 18,00 | 27,00 | 1,00 |
|  | 15,0 | 1 | 19,00 | 18,00 | 23,00 | 1,00 |
|  | 16,0 | 1 | 9,00 | 23,00 | 25,00 | 1,00 |
|  | 15,0 | 1 | 15,00 | 17,00 | 18,00 | 3,00 |
|  | 16,0 | 1 | 10,00 | 17,00 | 23,00 | 2,00 |
|  | 15,0 | 2 | 16,00 | 18,00 | 23,00 | 1,00 |
|  | 15,0 | 2 | 13,00 | 14,00 | 25,00 | 1,00 |
|  | 15,0 | 1 | 7,00 | 24,00 | 28,00 | 2,00 |
|  | 15,0 | 2 | 13,00 | 6,00 | 18,00 | 1,00 |
|  | 15,0 | 2 | 20,00 | 9,00 | 24,00 | 1,00 |
|  | 15,0 | 1 | 17,00 | 18,00 | 21,00 | 1,00 |
|  | 15,0 | 2 | 14,00 | 18,00 | 20,00 | 1,00 |
|  | 15,0 | 1 | 27,00 | 24,00 | 29,00 | 1,00 |
|  | 16,0 | 1 | 8,00 | 23,00 | 25,00 | 3,00 |
|  | 15,0 | 2 | 14,00 | 16,00 | 25,00 | 1,00 |
|  | 16,0 | 2 | 9,00 | 25,00 | 26,00 | 2,00 |
|  | 16,0 | 2 | 15,00 | 14,00 | 17,00 | 1,00 |
|  | 15,0 | 2 | 19,00 | 18,00 | 20,00 | 1,00 |
|  | 15,0 | 1 | 17,00 | 10,00 | 23,00 | 1,00 |
|  | 16,0 | 2 | 17,00 | 20,00 | 29,00 | 1,00 |
|  | 15,0 | 1 | 8,00 | 14,00 | 18,00 | 3,00 |
|  | 17,0 | 1 | 29,00 | 15,00 | 10,00 | 1,00 |
|  | 15,0 | 1 | 13,00 | 22,00 | 19,00 | 1,00 |
|  | 15,0 | 2 | 18,00 | 13,00 | 26,00 | 2,00 |
|  | 15,0 | 2 | 10,00 | 18,00 | 26,00 | 1,00 |
|  | 15,0 | 1 | 13,00 | 15,00 | 23,00 | 1,00 |
|  | 15,0 | 2 | 14,00 | 11,00 | 16,00 | 1,00 |
|  | 15,0 | 2 | 16,00 | 13,00 | 27,00 | 1,00 |
|  | 16,0 | 1 | 8,00 | 20,00 | 19,00 | 1,00 |
|  | 15,0 | 2 | 13,00 | 21,00 | 29,00 | 1,00 |
|  | 15,0 | 1 | 11,00 | 25,00 | 28,00 | 1,00 |
|  | 16,0 | 2 | 25,00 | 10,00 | 15,00 | 1,00 |
|  | 15,0 | 1 | 15,00 | 17,00 | 12,00 | 1,00 |
|  | 16,0 | 1 | 10,00 | 20,00 | 26,00 | 2,00 |
|  | 15,0 | 1 | 5,00 | 20,00 | 16,00 | 1,00 |
|  | 15,0 | 2 | 13,00 | 16,00 | 31,00 | 2,00 |
|  | 16,0 | 2 | 13,00 | 11,00 | 18,00 | 1,00 |
|  | 16,0 | 2 | 11,00 | 5,00 | 16,00 | 1,00 |
|  | 16,0 | 1 | 28,00 | 4,00 | 13,00 | 1,00 |
|  | 16,0 | 2 | 16,00 | 7,00 | 20,00 | 3,00 |
|  | 16,0 | 1 | 22,00 | 20,00 | 29,00 | 2,00 |
|  | 17,0 | 2 | 20,00 | 11,00 | 15,00 | 1,00 |
|  | 16,0 | 2 | 3,00 | 14,00 | 15,00 | 1,00 |
|  | 16,0 | 2 | 19,00 | 21,00 | 27,00 | 3,00 |
|  | 16,0 | 2 | 26,00 | 8,00 | 15,00 | 1,00 |
|  | 16,0 | 2 | 11,00 | 7,00 | 26,00 | 2,00 |
|  | 16,0 | 2 | 21,00 | 12,00 | 26,00 | 1,00 |
|  | 17,0 | 2 | 16,00 | 14,00 | 19,00 | 1,00 |
|  | 16,0 | 2 | 10,00 | 6,00 | 17,00 | 1,00 |
|  | 16,0 | 2 | 8,00 | 9,00 | 28,00 | 1,00 |
|  | 16,0 | 1 | 20,00 | 11,00 | 29,00 | 2,00 |
|  | 16,0 | 2 | 9,00 | 17,00 | 21,00 | 1,00 |
|  | 16,0 | 2 | 19,00 | 13,00 | 20,00 | 1,00 |
|  | 16,0 | 1 | 2,00 | 17,00 | 13,00 | 1,00 |
|  | 16,0 | 2 | 27,00 | 9,00 | 11,00 | 2,00 |
|  | 16,0 | 2 | 13,00 | 20,00 | 28,00 | 1,00 |
|  | 16,0 | 1 | 13,00 | 26,00 | 26,00 | 1,00 |
|  | 16,0 | 2 | 7,00 | 7,00 | 12,00 | 1,00 |
|  | 16,0 | 2 | 10,00 | 8,00 | 22,00 | 2,00 |
|  | 16,0 | 2 | 12,00 | 10,00 | 15,00 | 2,00 |
|  | 16,0 | 1 | 18,00 | 14,00 | 25,00 | 1,00 |
|  | 17,0 | 1 | 20,00 | 17,00 | 15,00 | 1,00 |
|  | 17,0 | 2 | 23,00 | 22,00 | 13,00 | 2,00 |
|  | 16,0 | 1 | 17,00 | 12,00 | 30,00 | 2,00 |
|  | 16,0 | 2 | 12,00 | 22,00 | 13,00 | 3,00 |
|  | 16,0 | 1 | 6,00 | 13,00 | 15,00 | 1,00 |
|  | 17,0 | 1 | 15,00 | 13,00 | 26,00 | 2,00 |
|  | 16,0 | 2 | 10,00 | 13,00 | 28,00 | 1,00 |
|  | 18,0 | 2 | 13,00 | 19,00 | 9,00 | 2,00 |
|  | 16,0 | 2 | 21,00 | 8,00 | 13,00 | 1,00 |
|  | 16,0 | 1 | 6,00 | 7,00 | 9,00 | 1,00 |
|  | 16,0 | 2 | 9,00 | ,00 | 13,00 | 1,00 |
|  | 16,0 | 2 | 21,00 | 9,00 | 16,00 | 2,00 |
|  | 18,0 | 1 | 16,00 | 28,00 | 25,00 | 1,00 |
|  | 18,0 | 1 | 11,00 | 24,00 | 27,00 | 3,00 |
|  | 17,0 | 2 | 13,00 | 6,00 | 20,00 | 1,00 |
|  | 16,0 | 2 | 21,00 | 16,00 | 17,00 | 1,00 |
|  | 16,0 | 1 | 7,00 | 16,00 | 24,00 | 2,00 |
|  | 16,0 | 1 | 9,00 | 12,00 | 16,00 | 1,00 |
|  | 16,0 | 2 | 11,00 | 22,00 | 31,00 | 1,00 |
|  | 16,0 | 2 | 14,00 | 16,00 | 17,00 | 1,00 |
|  | 16,0 | 1 | 12,00 | 24,00 | 25,00 | 1,00 |
|  | 16,0 | 2 | 12,00 | 7,00 | 20,00 | 1,00 |
|  | 17,0 | 2 | 17,00 | 17,00 | 21,00 | 1,00 |
|  | 18,0 | 1 | 20,00 | 22,00 | 21,00 | 1,00 |
|  | 16,0 | 2 | 20,00 | 16,00 | 24,00 | 1,00 |
|  | 16,0 | 1 | 13,00 | 22,00 | 27,00 | 1,00 |
|  | 16,0 | 2 | 10,00 | 13,00 | 20,00 | 1,00 |
|  | 16,0 | 2 | 23,00 | 8,00 | 24,00 | 1,00 |
|  | 16,0 | 1 | 16,00 | 5,00 | 20,00 | 1,00 |
|  | 18,0 | 1 | 21,00 | 16,00 | 19,00 | 1,00 |
|  | 17,0 | 2 | 25,00 | 11,00 | 28,00 | 1,00 |
|  | 17,0 | 1 | 21,00 | 16,00 | 15,00 | 2,00 |
|  | 17,0 | 1 | 12,00 | 21,00 | 16,00 | 1,00 |
|  | 17,0 | 2 | 20,00 | 14,00 | 18,00 | 2,00 |
|  | 17,0 | 1 | 25,00 | 28,00 | 13,00 | 1,00 |
|  | 17,0 | 2 | 15,00 | 15,00 | 19,00 | 1,00 |
|  | 17,0 | 1 | 7,00 | 14,00 | 25,00 | 1,00 |
|  | 17,0 | 1 | 17,00 | 6,00 | 24,00 | 2,00 |
|  | 18,0 | 2 | 17,00 | 19,00 | 17,00 | 1,00 |
|  | 17,0 | 1 | 24,00 | 22,00 | 26,00 | 1,00 |
|  | 18,0 | 2 | 22,00 | 16,00 | 17,00 | 2,00 |
|  | 18,0 | 1 | 15,00 | 11,00 | 11,00 | 1,00 |
|  | 17,0 | 2 | 13,00 | 9,00 | 20,00 | 1,00 |
|  | 17,0 | 2 | 15,00 | 9,00 | 22,00 | 1,00 |
|  | 18,0 | 2 | 13,00 | 17,00 | 19,00 | 2,00 |
|  | 17,0 | 2 | 17,00 | 9,00 | 13,00 | 1,00 |
|  | 18,0 | 1 | 18,00 | 20,00 | 26,00 | 1,00 |
|  | 17,0 | 1 | 12,00 | 14,00 | 20,00 | 1,00 |
|  | 17,0 | 2 | 21,00 | 22,00 | 32,00 | 1,00 |
|  | 17,0 | 1 | 21,00 | 17,00 | 23,00 | 2,00 |
|  | 17,0 | 1 | 28,00 | 20,00 | 32,00 | 2,00 |
|  | 17,0 | 2 | 17,00 | 27,00 | 15,00 | 1,00 |
|  | 17,0 | 2 | 26,00 | 14,00 | 12,00 | 1,00 |
|  | 18,0 | 2 | 26,00 | 8,00 | 13,00 | 1,00 |
|  | 17,0 | 2 | 16,00 | 19,00 | 18,00 | 1,00 |
|  | 18,0 | 1 | 10,00 | 10,00 | 14,00 | 2,00 |
|  | 18,0 | 2 | 13,00 | 13,00 | 16,00 | 1,00 |
|  | 17,0 | 2 | 14,00 | 22,00 | 21,00 | 1,00 |
|  | 17,0 | 2 | 17,00 | 12,00 | 32,00 | 2,00 |
|  | 17,0 | 2 | 17,00 | 21,00 | 25,00 | 1,00 |
|  | 17,0 | 2 | 9,00 | 28,00 | 20,00 | 1,00 |
|  | 18,0 | 2 | 9,00 | 14,00 | 22,00 | 1,00 |
|  | 18,0 | 1 | 19,00 | 15,00 | 17,00 | 2,00 |
|  | 17,0 | 2 | 18,00 | 10,00 | 20,00 | 1,00 |
|  | 17,0 | 2 | 22,00 | 9,00 | 15,00 | 1,00 |
|  | 17,0 | 2 | 30,00 | 19,00 | 30,00 | 2,00 |
|  | 17,0 | 1 | 8,00 | 26,00 | 21,00 | 1,00 |
|  | 17,0 | 1 | 24,00 | 21,00 | 30,00 | 2,00 |
|  | 17,0 | 2 | 5,00 | 10,00 | 21,00 | 1,00 |
|  | 16,0 | 1 | 30,00 | 21,00 | 28,00 | 1,00 |
|  | 17,0 | 2 | 11,00 | 9,00 | 14,00 | 2,00 |
|  | 17,0 | 2 | 20,00 | 9,00 | 13,00 | 2,00 |
|  | 17,0 | 1 | 19,00 | 6,00 | 15,00 | 1,00 |
|  | 18,0 | 1 | 12,00 | 15,00 | 24,00 | 1,00 |
|  | 18,0 | 2 | 12,00 | 23,00 | 26,00 | 1,00 |
|  | 18,0 | 1 | 20,00 | 23,00 | 21,00 | 2,00 |
|  | 17,0 | 2 | 28,00 | 19,00 | 8,00 | 2,00 |
|  | 17,0 | 2 | 12,00 | 20,00 | 25,00 | 2,00 |
|  | 17,0 | 1 | 11,00 | 14,00 | 28,00 | 2,00 |
|  | 18,0 | 2 | 17,00 | 11,00 | 16,00 | 1,00 |
|  | 18,0 | 1 | 9,00 | 23,00 | 30,00 | 2,00 |
|  | 17,0 | 1 | 29,00 | 22,00 | 15,00 | 2,00 |
|  | 17,0 | 2 | 11,00 | 13,00 | 3,00 | 2,00 |
|  | 18,0 | 2 | 25,00 | 14,00 | 20,00 | 1,00 |
|  | 18,0 | 2 | 5,00 | 11,00 | 22,00 | 3,00 |
|  | 18,0 | 1 | 13,00 | 22,00 | 28,00 | 2,00 |
|  | 18,0 | 1 | 14,00 | 11,00 | 21,00 | 1,00 |
|  | 18,0 | 1 | 17,00 | 19,00 | 21,00 | 2,00 |
|  | 12,0 | 1 | 9,00 | 11,00 | 29,00 | 1,00 |
|  | 13,0 | 2 | 23,00 | 30,00 | 26,00 | 1,00 |
|  | 12,0 | 1 | 31,00 | 28,00 | 19,00 | 1,00 |
|  | 12,0 | 2 | 16,00 | 23,00 | 24,00 | 1,00 |
|  | 13,0 | 1 | 22,00 | 19,00 | 27,00 | 1,00 |
|  | 13,0 | 2 | 27,00 | 25,00 | 28,00 | 3,00 |
|  | 12,0 | 2 | 13,00 | 6,00 | 13,00 | 1,00 |
|  | 12,0 | 1 | 16,00 | 20,00 | 12,00 | 1,00 |
|  | 12,0 | 1 | 10,00 | 14,00 | 17,00 | 1,00 |
|  | 12,0 | 1 | 28,00 | 16,00 | 30,00 | 2,00 |
|  | 12,0 | 1 | 14,00 | 7,00 | 16,00 | 2,00 |
|  | 12,0 | 2 | 16,00 | 20,00 | 25,00 | 3,00 |
|  | 12,0 | 2 | 30,00 | 14,00 | 17,00 | 1,00 |
|  | 12,0 | 2 | 22,00 | 17,00 | 18,00 | 1,00 |
|  | 12,0 | 2 | 23,00 | 15,00 | 32,00 | 1,00 |
|  | 12,0 | 1 | 8,00 | 17,00 | 26,00 | 3,00 |
|  | 12,0 | 1 | 9,00 | 8,00 | 11,00 | 3,00 |
|  | 12,0 | 1 | 4,00 | 8,00 | 8,00 | 3,00 |
|  | 12,0 | 2 | 25,00 | 23,00 | 17,00 | 1,00 |
|  | 12,0 | 2 | 24,00 | 18,00 | 25,00 | 1,00 |
|  | 12,0 | 2 | 9,00 | 15,00 | 24,00 | 3,00 |
|  | 12,0 | 1 | 14,00 | 13,00 | 22,00 | 3,00 |
|  | 12,0 | 2 | 8,00 | 17,00 | 25,00 | 3,00 |
|  | 13,0 | 1 | 27,00 | 28,00 | 18,00 | 2,00 |
|  | 12,0 | 1 | 20,00 | 22,00 | 25,00 | 2,00 |
|  | 12,0 | 2 | 14,00 | 14,00 | 27,00 | 1,00 |
|  | 13,0 | 1 | 28,00 | 24,00 | 29,00 | 3,00 |
|  | 12,0 | 2 | 29,00 | 13,00 | 29,00 | 2,00 |
|  | 12,0 | 2 | 2,00 | 15,00 | 13,00 | 3,00 |
|  | 12,0 | 2 | 28,00 | 24,00 | 27,00 | 1,00 |
|  | 12,0 | 1 | 16,00 | 13,00 | 32,00 | 1,00 |
|  | 12,0 | 2 | 24,00 | 13,00 | 27,00 | 1,00 |
|  | 12,0 | 2 | 15,00 | 21,00 | 31,00 | 3,00 |
|  | 13,0 | 1 | 16,00 | 14,00 | 18,00 | 2,00 |
|  | 13,0 | 2 | 9,00 | 16,00 | 25,00 | 1,00 |
|  | 12,0 | 2 | 25,00 | 26,00 | 28,00 | 3,00 |
|  | 12,0 | 2 | 10,00 | 17,00 | 27,00 | 1,00 |
|  | 13,0 | 1 | 23,00 | 27,00 | 29,00 | 1,00 |
|  | 13,0 | 2 | 26,00 | 28,00 | 27,00 | 2,00 |
|  | 12,0 | 2 | 12,00 | 9,00 | 16,00 | 1,00 |
|  | 12,0 | 1 | 13,00 | 19,00 | 26,00 | 1,00 |
|  | 13,0 | 1 | 15,00 | 13,00 | 18,00 | 1,00 |
|  | 12,0 | 1 | 5,00 | 9,00 | 13,00 | 1,00 |
|  | 12,0 | 2 | 8,00 | 4,00 | 8,00 | 3,00 |
|  | 12,0 | 1 | 28,00 | 27,00 | 24,00 | 1,00 |
|  | 12,0 | 2 | 16,00 | 21,00 | 19,00 | 3,00 |
|  | 12,0 | 1 | 4,00 | 16,00 | 13,00 | 3,00 |
|  | 12,0 | 2 | 10,00 | 14,00 | 22,00 | 1,00 |
|  | 12,0 | 2 | 25,00 | 21,00 | 31,00 | 1,00 |
|  | 12,0 | 1 | 5,00 | 14,00 | 16,00 | 1,00 |
|  | 12,0 | 1 | 18,00 | 17,00 | 17,00 | 1,00 |
|  | 12,0 | 2 | 18,00 | 15,00 | 22,00 | 2,00 |
|  | 12,0 | 1 | 17,00 | 15,00 | 25,00 | 2,00 |
|  | 12,0 | 2 | 13,00 | 2,00 | 2,00 | 2,00 |
|  | 12,0 | 1 | 17,00 | 10,00 | 22,00 | 3,00 |
|  | 12,0 | 1 | 20,00 | 16,00 | 24,00 | 1,00 |
|  | 13,0 | 2 | 6,00 | 4,00 | 8,00 | 1,00 |
|  | 13,0 | 2 | 29,00 | 30,00 | 32,00 | 3,00 |
|  | 12,0 | 2 | 15,00 | 11,00 | 21,00 | 1,00 |
|  | 13,0 | 2 | 14,00 | 25,00 | 26,00 | 3,00 |
|  | 13,0 | 1 | 16,00 | 22,00 | 26,00 | 3,00 |
|  | 12,0 | 2 | 24,00 | 24,00 | 26,00 | 1,00 |
|  | 12,0 | 1 | 19,00 | 10,00 | 26,00 | 1,00 |
|  | 12,0 | 2 | 24,00 | 25,00 | 26,00 | 1,00 |
|  | 13,0 | 2 | 23,00 | 21,00 | 32,00 | 2,00 |
|  | 12,0 | 1 | 32,00 | 16,00 | 1,00 | 1,00 |
|  | 12,0 | 2 | 16,00 | 23,00 | 28,00 | 1,00 |
|  | 12,0 | 2 | 24,00 | 21,00 | 27,00 | 1,00 |
|  | 12,0 | 1 | 27,00 | 31,00 | 29,00 | 1,00 |
|  | 12,0 | 1 | 26,00 | 29,00 | 32,00 | 2,00 |
|  | 12,0 | 2 | 20,00 | 19,00 | 32,00 | 2,00 |
|  | 14,0 | 1 | 23,00 | 23,00 | 30,00 | 1,00 |
|  | 13,0 | 2 | 25,00 | 21,00 | 32,00 | 3,00 |
|  | 13,0 | 2 | 8,00 | 8,00 | 10,00 | 1,00 |
|  | 13,0 | 1 | 25,00 | 22,00 | 28,00 | 3,00 |
|  | 13,0 | 2 | 15,00 | 6,00 | 32,00 | 1,00 |
|  | 13,0 | 2 | 9,00 | 18,00 | 27,00 | 1,00 |
|  | 13,0 | 2 | 22,00 | 31,00 | 32,00 | 1,00 |
|  | 14,0 | 2 | 13,00 | 10,00 | 25,00 | 2,00 |
|  | 13,0 | 1 | 10,00 | 22,00 | 17,00 | 2,00 |
|  | 13,0 | 1 | 16,00 | 16,00 | 23,00 | 1,00 |
|  | 13,0 | 2 | 9,00 | 12,00 | 17,00 | 1,00 |
|  | 14,0 | 2 | 12,00 | 8,00 | 8,00 | 2,00 |
|  | 14,0 | 2 | 26,00 | 22,00 | 30,00 | 2,00 |
|  | 15,0 | 1 | 25,00 | 21,00 | 27,00 | 1,00 |
|  | 13,0 | 2 | 4,00 | 7,00 | 20,00 | 1,00 |
|  | 13,0 | 2 | 27,00 | 20,00 | 26,00 | 2,00 |
|  | 13,0 | 1 | 8,00 | 7,00 | 17,00 | 3,00 |
|  | 13,0 | 2 | 25,00 | 29,00 | 31,00 | 1,00 |
|  | 14,0 | 2 | 24,00 | 26,00 | 17,00 | 2,00 |
|  | 13,0 | 2 | 9,00 | 2,00 | 8,00 | 1,00 |
|  | 14,0 | 2 | 9,00 | 27,00 | 29,00 | 1,00 |
|  | 14,0 | 1 | 9,00 | 20,00 | 27,00 | 1,00 |
|  | 14,0 | 1 | 18,00 | 23,00 | 23,00 | 1,00 |
|  | 13,0 | 2 | 23,00 | 24,00 | 28,00 | 1,00 |
|  | 14,0 | 2 | 19,00 | 12,00 | 27,00 | 3,00 |
|  | 14,0 | 1 | 15,00 | 22,00 | 22,00 | 1,00 |
|  | 14,0 | 1 | 15,00 | 7,00 | 9,00 | 1,00 |
|  | 14,0 | 1 | 10,00 | 12,00 | 13,00 | 1,00 |
|  | 13,0 | 1 | 23,00 | 18,00 | 27,00 | 1,00 |
|  | 13,0 | 2 | 15,00 | 16,00 | 21,00 | 1,00 |
|  | 14,0 | 2 | 21,00 | 27,00 | 27,00 | 1,00 |
|  | 13,0 | 1 | 15,00 | 18,00 | 19,00 | 1,00 |
|  | 14,0 | 2 | 29,00 | 14,00 | 19,00 | 1,00 |
|  | 13,0 | 1 | 12,00 | 14,00 | 22,00 | 1,00 |
|  | 13,0 | 1 | 24,00 | 25,00 | 26,00 | 1,00 |
|  | 14,0 | 1 | 14,00 | 21,00 | 24,00 | 2,00 |
|  | 13,0 | 2 | 26,00 | 20,00 | 22,00 | 2,00 |
|  | 14,0 | 1 | 9,00 | 12,00 | 14,00 | 1,00 |
|  | 13,0 | 1 | 12,00 | 23,00 | 29,00 | 1,00 |
|  | 13,0 | 1 | 29,00 | 15,00 | 10,00 | 1,00 |
|  | 13,0 | 1 | 8,00 | 15,00 | 13,00 | 1,00 |
|  | 14,0 | 1 | 10,00 | 18,00 | 27,00 | 1,00 |
|  | 13,0 | 2 | 25,00 | 20,00 | 15,00 | 1,00 |
|  | 14,0 | 2 | 13,00 | 18,00 | 26,00 | 1,00 |
|  | 13,0 | 1 | 16,00 | 23,00 | 28,00 | 2,00 |
|  | 13,0 | 2 | 7,00 | 12,00 | 23,00 | 2,00 |
|  | 14,0 | 2 | 17,00 | 15,00 | 23,00 | 1,00 |
|  | 13,0 | 2 | 25,00 | 22,00 | 28,00 | 1,00 |
|  | 13,0 | 1 | 11,00 | 14,00 | 19,00 | 1,00 |
|  | 14,0 | 1 | 3,00 | 17,00 | 23,00 | 1,00 |
|  | 14,0 | 1 | 2,00 | 22,00 | 18,00 | 1,00 |
|  | 13,0 | 2 | 27,00 | 18,00 | 32,00 | 3,00 |
|  | 14,0 | 2 | 11,00 | 14,00 | 26,00 | 1,00 |
|  | 14,0 | 1 | 21,00 | 23,00 | 25,00 | 1,00 |
|  | 14,0 | 2 | 11,00 | 21,00 | 22,00 | 1,00 |
|  | 13,0 | 2 | 13,00 | 10,00 | 28,00 | 1,00 |
|  | 13,0 | 1 | 16,00 | 14,00 | 23,00 | 1,00 |
|  | 16,0 | 2 | 30,00 | 13,00 | 26,00 | 1,00 |
|  | 13,0 | 1 | 21,00 | 17,00 | 24,00 | 2,00 |
|  | 13,0 | 1 | 14,00 | 16,00 | 16,00 | 1,00 |
|  | 14,0 | 1 | 21,00 | 19,00 | 9,00 | 1,00 |
|  | 13,0 | 1 | 7,00 | 7,00 | 16,00 | 3,00 |
|  | 13,0 | 2 | 27,00 | 28,00 | 32,00 | 2,00 |
|  | 14,0 | 2 | 17,00 | 21,00 | 29,00 | 1,00 |
|  | 13,0 | 2 | 17,00 | 14,00 | 23,00 | 1,00 |
|  | 13,0 | 1 | 9,00 | 8,00 | 18,00 | 3,00 |
|  | 13,0 | 2 | 8,00 | 12,00 | 17,00 | 1,00 |
|  | 13,0 | 2 | 10,00 | 19,00 | 18,00 | 3,00 |
|  | 13,0 | 1 | 5,00 | 28,00 | 13,00 | 1,00 |
|  | 13,0 | 1 | 14,00 | 13,00 | 19,00 | 1,00 |
|  | 13,0 | 2 | 12,00 | 4,00 | 12,00 | 1,00 |
|  | 13,0 | 1 | ,00 | ,00 | 26,00 | 2,00 |
|  | 13,0 | 1 | 5,00 | 5,00 | 25,00 | 1,00 |
|  | 13,0 | 1 | 13,00 | 16,00 | 26,00 | 1,00 |
|  | 13,0 | 1 | 10,00 | 14,00 | 17,00 | 3,00 |
|  | 13,0 | 1 | 13,00 | 14,00 | 18,00 | 1,00 |
|  | 13,0 | 2 | 21,00 | 22,00 | 14,00 | 1,00 |
|  | 13,0 | 1 | 9,00 | 14,00 | 16,00 | 1,00 |
|  | 13,0 | 1 | 17,00 | 23,00 | 26,00 | 2,00 |
|  | 13,0 | 2 | 16,00 | 13,00 | 22,00 | 1,00 |
|  | 14,0 | 1 | 22,00 | 24,00 | 26,00 | 2,00 |
|  | 14,0 | 2 | 16,00 | 13,00 | 20,00 | 1,00 |
|  | 13,0 | 2 | 23,00 | 11,00 | 13,00 | 1,00 |
|  | 14,0 | 2 | 22,00 | 7,00 | 25,00 | 2,00 |
|  | 14,0 | 2 | 12,00 | 8,00 | 29,00 | 3,00 |
|  | 13,0 | 2 | 12,00 | 10,00 | 18,00 | 1,00 |
|  | 13,0 | 1 | 8,00 | 22,00 | 21,00 | 3,00 |
|  | 13,0 | 2 | 10,00 | 17,00 | 25,00 | 1,00 |
|  | 13,0 | 2 | 5,00 | 1,00 | 5,00 | 1,00 |
|  | 13,0 | 2 | 24,00 | 15,00 | 31,00 | 1,00 |
|  | 13,0 | 1 | 23,00 | 27,00 | 16,00 | 1,00 |
|  | 13,0 | 1 | 9,00 | 8,00 | 13,00 | 1,00 |
|  | 14,0 | 1 | 11,00 | 25,00 | 12,00 | 1,00 |
|  | 14,0 | 2 | 19,00 | 18,00 | 23,00 | 1,00 |
|  | 14,0 | 2 | 14,00 | 12,00 | 27,00 | 1,00 |
|  | 13,0 | 1 | 13,00 | 17,00 | 30,00 | 1,00 |
|  | 13,0 | 1 | 14,00 | 13,00 | 26,00 | 1,00 |
|  | 14,0 | 1 | 11,00 | 12,00 | 2,00 | 1,00 |
|  | 14,0 | 2 | 13,00 | 14,00 | 18,00 | 1,00 |
|  | 13,0 | 1 | 1,00 | 11,00 | 2,00 | 1,00 |
|  | 13,0 | 2 | ,00 | 4,00 | 5,00 | 2,00 |
|  | 13,0 | 2 | 10,00 | 17,00 | 21,00 | 1,00 |
|  | 14,0 | 2 | 12,00 | 9,00 | 24,00 | 1,00 |
|  | 13,0 | 1 | 16,00 | 12,00 | 15,00 | 3,00 |
|  | 13,0 | 2 | 20,00 | 8,00 | 18,00 | 1,00 |
|  | 14,0 | 1 | 29,00 | 26,00 | 14,00 | 1,00 |
|  | 18,0 | 2 | 17,00 | 19,00 | 15,00 | 1,00 |
|  | 14,0 | 2 | 23,00 | 5,00 | 13,00 | 2,00 |
|  | 15,0 | 1 | 10,00 | 21,00 | 29,00 | 1,00 |
|  | 15,0 | 1 | 18,00 | 18,00 | 28,00 | 2,00 |
|  | 16,0 | 1 | 5,00 | 19,00 | 27,00 | 2,00 |
|  | 14,0 | 2 | 24,00 | 18,00 | 29,00 | 1,00 |
|  | 15,0 | 1 | 27,00 | 17,00 | 23,00 | 1,00 |
|  | 14,0 | 2 | 29,00 | 25,00 | 9,00 | 2,00 |
|  | 14,0 | 1 | 18,00 | 20,00 | 22,00 | 3,00 |
|  | 14,0 | 2 | 14,00 | 17,00 | 32,00 | 1,00 |
|  | 15,0 | 2 | 26,00 | 19,00 | 28,00 | 2,00 |
|  | 15,0 | 1 | 8,00 | 12,00 | 20,00 | 1,00 |
|  | 15,0 | 2 | 29,00 | 16,00 | 8,00 | 1,00 |
|  | 14,0 | 2 | 15,00 | 14,00 | 25,00 | 2,00 |
|  | 14,0 | 2 | 17,00 | 15,00 | 14,00 | 1,00 |
|  | 14,0 | 2 | 13,00 | 7,00 | 25,00 | 1,00 |
|  | 15,0 | 2 | 20,00 | 23,00 | 32,00 | 2,00 |
|  | 14,0 | 1 | 5,00 | 10,00 | 15,00 | 1,00 |
|  | 14,0 | 1 | 15,00 | 19,00 | 22,00 | 1,00 |
|  | 14,0 | 1 | 20,00 | 27,00 | 31,00 | 1,00 |
|  | 14,0 | 1 | 21,00 | 13,00 | 20,00 | 2,00 |
|  | 15,0 | 2 | 20,00 | 22,00 | 26,00 | 1,00 |
|  | 14,0 | 1 | 15,00 | 20,00 | 25,00 | 1,00 |
|  | 14,0 | 1 | 4,00 | 8,00 | 12,00 | 3,00 |
|  | 14,0 | 1 | 10,00 | 19,00 | 30,00 | 3,00 |
|  | 14,0 | 1 | 12,00 | 30,00 | 32,00 | 1,00 |
|  | 15,0 | 1 | 21,00 | 18,00 | 22,00 | 2,00 |
|  | 14,0 | 1 | 8,00 | 11,00 | 16,00 | 1,00 |
|  | 15,0 | 1 | 15,00 | 18,00 | 19,00 | 1,00 |
|  | 14,0 | 2 | 19,00 | 12,00 | 23,00 | 1,00 |
|  | 14,0 | 2 | 7,00 | 17,00 | 27,00 | 3,00 |
|  | 14,0 | 2 | 26,00 | 28,00 | 31,00 | 1,00 |
|  | 14,0 | 2 | 5,00 | 5,00 | 25,00 | 2,00 |
|  | 14,0 | 2 | 20,00 | 22,00 | 32,00 | 3,00 |
|  | 15,0 | 1 | 18,00 | 20,00 | 26,00 | 1,00 |
|  | 15,0 | 1 | 5,00 | 13,00 | 22,00 | 3,00 |
|  | 14,0 | 1 | 11,00 | 14,00 | 24,00 | 1,00 |
|  | 15,0 | 1 | 20,00 | 19,00 | 32,00 | 1,00 |
|  | 15,0 | 1 | 23,00 | 26,00 | 25,00 | 1,00 |
|  | 15,0 | 2 | 16,00 | 6,00 | 15,00 | 1,00 |
|  | 15,0 | 1 | 21,00 | 21,00 | 30,00 | 2,00 |
|  | 15,0 | 2 | 12,00 | 29,00 | 25,00 | 3,00 |
|  | 14,0 | 2 | 5,00 | 15,00 | 24,00 | 3,00 |
|  | 14,0 | 1 | 19,00 | 16,00 | 14,00 | 2,00 |
|  | 14,0 | 1 | 10,00 | 17,00 | 9,00 | 1,00 |
|  | 15,0 | 2 | 16,00 | 18,00 | 14,00 | 2,00 |
|  | 14,0 | 1 | 18,00 | 21,00 | 22,00 | 2,00 |
|  | 15,0 | 2 | 17,00 | 20,00 | 32,00 | 2,00 |
|  | 15,0 | 2 | 22,00 | 8,00 | 19,00 | 1,00 |
|  | 14,0 | 2 | 18,00 | 13,00 | 18,00 | 1,00 |
|  | 14,0 | 2 | 7,00 | 9,00 | 21,00 | 1,00 |
|  | 15,0 | 2 | 12,00 | 22,00 | 24,00 | 2,00 |
|  | 15,0 | 2 | 21,00 | 21,00 | 27,00 | 2,00 |
|  | 14,0 | 2 | 8,00 | 16,00 | 7,00 | 1,00 |
|  | 14,0 | 1 | 23,00 | 23,00 | 24,00 | 2,00 |
|  | 14,0 | 1 | 18,00 | 23,00 | 28,00 | 2,00 |
|  | 15,0 | 1 | 6,00 | 3,00 | 12,00 | 2,00 |
|  | 14,0 | 1 | 23,00 | 25,00 | 13,00 | 1,00 |
|  | 15,0 | 2 | 14,00 | 14,00 | 24,00 | 1,00 |
|  | 14,0 | 2 | 15,00 | 23,00 | 25,00 | 1,00 |
|  | 15,0 | 1 | 28,00 | 29,00 | 32,00 | 1,00 |
|  | 15,0 | 1 | 25,00 | 21,00 | 28,00 | 1,00 |
|  | 14,0 | 1 | 9,00 | 13,00 | 25,00 | 1,00 |
|  | 15,0 | 1 | 1,00 | 13,00 | 17,00 | 1,00 |
|  | 16,0 | 2 | 9,00 | 4,00 | 20,00 | 1,00 |
|  | 15,0 | 1 | 24,00 | 31,00 | 30,00 | 2,00 |
|  | 14,0 | 2 | 12,00 | 4,00 | 8,00 | 1,00 |
|  | 15,0 | 1 | 14,00 | 13,00 | 20,00 | 1,00 |
|  | 14,0 | 1 | 14,00 | 16,00 | 22,00 | 2,00 |
|  | 15,0 | 2 | 15,00 | 13,00 | 22,00 | 1,00 |
|  | 14,0 | 2 | 17,00 | 27,00 | 29,00 | 1,00 |
|  | 15,0 | 1 | 14,00 | 16,00 | 27,00 | 3,00 |
|  | 14,0 | 2 | 24,00 | 19,00 | 28,00 | 2,00 |
|  | 14,0 | 1 | 12,00 | 23,00 | 22,00 | 1,00 |
|  | 14,0 | 1 | 14,00 | 18,00 | 24,00 | 2,00 |
|  | 15,0 | 2 | 17,00 | 18,00 | 23,00 | 1,00 |
|  | 15,0 | 2 | 21,00 | 25,00 | 23,00 | 2,00 |
|  | 15,0 | 2 | 18,00 | 14,00 | 14,00 | 2,00 |
|  | 14,0 | 1 | 2,00 | 8,00 | 12,00 | 1,00 |
|  | 14,0 | 2 | 9,00 | 5,00 | 4,00 | 1,00 |
|  | 15,0 | 1 | 20,00 | 16,00 | 21,00 | 1,00 |
|  | 14,0 | 2 | 26,00 | 20,00 | 23,00 | 2,00 |
|  | 14,0 | 2 | 13,00 | 17,00 | 12,00 | 2,00 |
|  | 14,0 | 2 | 19,00 | 16,00 | 17,00 | 1,00 |
|  | 15,0 | 2 | 22,00 | 15,00 | 15,00 | 2,00 |
|  | 15,0 | 2 | 9,00 | 15,00 | 19,00 | 3,00 |
|  | 16,0 | 2 | 16,00 | 17,00 | 30,00 | 3,00 |
|  | 15,0 | 1 | 25,00 | 23,00 | 31,00 | 1,00 |
|  | 16,0 | 1 | 10,00 | 11,00 | 24,00 | 1,00 |
|  | 15,0 | 2 | 8,00 | 8,00 | 30,00 | 2,00 |
|  | 15,0 | 2 | 8,00 | 13,00 | 16,00 | 3,00 |
|  | 15,0 | 1 | 22,00 | 19,00 | 16,00 | 1,00 |
|  | 15,0 | 1 | 15,00 | 20,00 | 16,00 | 2,00 |
|  | 15,0 | 1 | 15,00 | 23,00 | 25,00 | 2,00 |
|  | 16,0 | 1 | 24,00 | 20,00 | 23,00 | 1,00 |
|  | 15,0 | 1 | 18,00 | 17,00 | 20,00 | 2,00 |
|  | 16,0 | 2 | 7,00 | 17,00 | 25,00 | 1,00 |
|  | 15,0 | 2 | 28,00 | 8,00 | 14,00 | 1,00 |
|  | 15,0 | 1 | 4,00 | 11,00 | 20,00 | 1,00 |
|  | 16,0 | 2 | 23,00 | 14,00 | 21,00 | 1,00 |
|  | 15,0 | 2 | 18,00 | 11,00 | 22,00 | 2,00 |
|  | 16,0 | 2 | 22,00 | 24,00 | 19,00 | 2,00 |
|  | 15,0 | 2 | 16,00 | 6,00 | 10,00 | 2,00 |
|  | 16,0 | 2 | 20,00 | 20,00 | 21,00 | 3,00 |
|  | 15,0 | 1 | 17,00 | 22,00 | 26,00 | 1,00 |
|  | 16,0 | 2 | 14,00 | 11,00 | 31,00 | 3,00 |
|  | 15,0 | 2 | 24,00 | 9,00 | 14,00 | 2,00 |
|  | 15,0 | 2 | 15,00 | 26,00 | 26,00 | 1,00 |
|  | 15,0 | 2 | 18,00 | 24,00 | 30,00 | 1,00 |
|  | 16,0 | 1 | 25,00 | 18,00 | 15,00 | 2,00 |
|  | 15,0 | 2 | 19,00 | 11,00 | 29,00 | 1,00 |
|  | 15,0 | 1 | 17,00 | 13,00 | 19,00 | 1,00 |
|  | 15,0 | 2 | 20,00 | 11,00 | 28,00 | 1,00 |
|  | 15,0 | 2 | 8,00 | 8,00 | 27,00 | 1,00 |
|  | 16,0 | 2 | 9,00 | 23,00 | 32,00 | 1,00 |
|  | 18,0 | 1 | 16,00 | 18,00 | 18,00 | 1,00 |
|  | 16,0 | 1 | 11,00 | 11,00 | 15,00 | 1,00 |
|  | 17,0 | 1 | 7,00 | 14,00 | 20,00 | 3,00 |
|  | 17,0 | 1 | 22,00 | 14,00 | 16,00 | 2,00 |
|  | 16,0 | 2 | 21,00 | 18,00 | 9,00 | 2,00 |
|  | 16,0 | 2 | 10,00 | 19,00 | 27,00 | 1,00 |
|  | 16,0 | 2 | 21,00 | 23,00 | 27,00 | 1,00 |
|  | 16,0 | 1 | 11,00 | 15,00 | 27,00 | 1,00 |
|  | 16,0 | 1 | 5,00 | 10,00 | 20,00 | 1,00 |
|  | 16,0 | 2 | 16,00 | 11,00 | 29,00 | 1,00 |
|  | 16,0 | 2 | 10,00 | 9,00 | 20,00 | 1,00 |
|  | 16,0 | 1 | 11,00 | 23,00 | 31,00 | 1,00 |
|  | 16,0 | 1 | 23,00 | 14,00 | 26,00 | 1,00 |
|  | 16,0 | 2 | 24,00 | 21,00 | 21,00 | 1,00 |
|  | 17,0 | 2 | 8,00 | 25,00 | 27,00 | 1,00 |
|  | 17,0 | 1 | 18,00 | 24,00 | 21,00 | 2,00 |
|  | 16,0 | 2 | 21,00 | 26,00 | 30,00 | 2,00 |
|  | 18,0 | 2 | 20,00 | 25,00 | 19,00 | 1,00 |
|  | 16,0 | 1 | 18,00 | 23,00 | 19,00 | 2,00 |
|  | 16,0 | 1 | 20,00 | 13,00 | 19,00 | 1,00 |
|  | 16,0 | 2 | 18,00 | 13,00 | 25,00 | 1,00 |
|  | 16,0 | 1 | 18,00 | 15,00 | 15,00 | 1,00 |
|  | 17,0 | 2 | 20,00 | 16,00 | 19,00 | 1,00 |
|  | 16,0 | 1 | 10,00 | 17,00 | 11,00 | 2,00 |
|  | 16,0 | 1 | 18,00 | 16,00 | 24,00 | 1,00 |
|  | 16,0 | 2 | 21,00 | 22,00 | 24,00 | 2,00 |
|  | 17,0 | 2 | 22,00 | 23,00 | 30,00 | 1,00 |
|  | 16,0 | 1 | 6,00 | 19,00 | 22,00 | 1,00 |
|  | 17,0 | 2 | 11,00 | 8,00 | 18,00 | 1,00 |
|  | 16,0 | 1 | 8,00 | 14,00 | 18,00 | 1,00 |
|  | 17,0 | 2 | 17,00 | 15,00 | 12,00 | 2,00 |
|  | 17,0 | 1 | 18,00 | 21,00 | 24,00 | 1,00 |
|  | 16,0 | 1 | 28,00 | 28,00 | 26,00 | 2,00 |
|  | 17,0 | 1 | 24,00 | 14,00 | 25,00 | 1,00 |
|  | 16,0 | 1 | 19,00 | 25,00 | 20,00 | 1,00 |
|  | 16,0 | 1 | 27,00 | 16,00 | 16,00 | 1,00 |
|  | 17,0 | 2 | 22,00 | 20,00 | 22,00 | 1,00 |
|  | 18,0 | 2 | 25,00 | 13,00 | 28,00 | 2,00 |
|  | 16,0 | 1 | 18,00 | 9,00 | 23,00 | 1,00 |
|  | 16,0 | 2 | 12,00 | 12,00 | 30,00 | 1,00 |
|  | 18,0 | 2 | 25,00 | 8,00 | 23,00 | 1,00 |
|  | 17,0 | 1 | 24,00 | 23,00 | 24,00 | 1,00 |
|  | 16,0 | 2 | 15,00 | 19,00 | 9,00 | 2,00 |
|  | 17,0 | 1 | 12,00 | 22,00 | 26,00 | 1,00 |
|  | 17,0 | 2 | 15,00 | 17,00 | 26,00 | 1,00 |
|  | 17,0 | 1 | 5,00 | 32,00 | 15,00 | 1,00 |
|  | 16,0 | 2 | 10,00 | 9,00 | 12,00 | 1,00 |
|  | 17,0 | 1 | 15,00 | 27,00 | 25,00 | 3,00 |
|  | 17,0 | 1 | 13,00 | 20,00 | 23,00 | 1,00 |
|  | 16,0 | 2 | 21,00 | 14,00 | 23,00 | 1,00 |
|  | 16,0 | 1 | 9,00 | 6,00 | 6,00 | 1,00 |
|  | 16,0 | 2 | 30,00 | 24,00 | 25,00 | 1,00 |
|  | 16,0 | 1 | 15,00 | 11,00 | 21,00 | 2,00 |
|  | 16,0 | 2 | 24,00 | 19,00 | 25,00 | 1,00 |
|  | 17,0 | 2 | 22,00 | 18,00 | 26,00 | 1,00 |
|  | 16,0 | 1 | 20,00 | 25,00 | 32,00 | 1,00 |
|  | 16,0 | 2 | 28,00 | 24,00 | 21,00 | 1,00 |
|  | 16,0 | 2 | 9,00 | 15,00 | 26,00 | 3,00 |
|  | 16,0 | 2 | 32,00 | 20,00 | 28,00 | 1,00 |
|  | 16,0 | 1 | 14,00 | 10,00 | 25,00 | 3,00 |
|  | 17,0 | 1 | 18,00 | 19,00 | 18,00 | 1,00 |
|  | 17,0 | 2 | 28,00 | 26,00 | 11,00 | 1,00 |
|  | 16,0 | 2 | 29,00 | 15,00 | 22,00 | 1,00 |
|  | 17,0 | 1 | 28,00 | 15,00 | 28,00 | 2,00 |
|  | 16,0 | 2 | 31,00 | 14,00 | 15,00 | 2,00 |
|  | 17,0 | 1 | 14,00 | 28,00 | 29,00 | 1,00 |
|  | 16,0 | 2 | 18,00 | 17,00 | 28,00 | 1,00 |
|  | 16,0 | 2 | 23,00 | 18,00 | 28,00 | 3,00 |
|  | 16,0 | 2 | 19,00 | 15,00 | 23,00 | 1,00 |
|  | 16,0 | 1 | 23,00 | 16,00 | 22,00 | 1,00 |
|  | 16,0 | 2 | 22,00 | 25,00 | 22,00 | 1,00 |
|  | 17,0 | 2 | 14,00 | 24,00 | 27,00 | 2,00 |
|  | 17,0 | 1 | 29,00 | 27,00 | 10,00 | 2,00 |
|  | 17,0 | 2 | 21,00 | 15,00 | 9,00 | 1,00 |
|  | 16,0 | 2 | 28,00 | 19,00 | 29,00 | 2,00 |
|  | 16,0 | 2 | 26,00 | 7,00 | 25,00 | 1,00 |
|  | 17,0 | 2 | 11,00 | 12,00 | 5,00 | 1,00 |
|  | 17,0 | 2 | 12,00 | 15,00 | 15,00 | 1,00 |
|  | 18,0 | 2 | 10,00 | 13,00 | 25,00 | 1,00 |
|  | 17,0 | 2 | 16,00 | 13,00 | 30,00 | 1,00 |
|  | 17,0 | 1 | 17,00 | 8,00 | 10,00 | 2,00 |
|  | 17,0 | 2 | 8,00 | 6,00 | 16,00 | 1,00 |
|  | 17,0 | 2 | 22,00 | 16,00 | 10,00 | 3,00 |
|  | 17,0 | 2 | 30,00 | 12,00 | 23,00 | 2,00 |
|  | 18,0 | 1 | 13,00 | 12,00 | 22,00 | 1,00 |
|  | 17,0 | 1 | 8,00 | 10,00 | 16,00 | 1,00 |
|  | 18,0 | 1 | 27,00 | 25,00 | 32,00 | 1,00 |
|  | 17,0 | 1 | 14,00 | 14,00 | 9,00 | 1,00 |
|  | 17,0 | 1 | 24,00 | 25,00 | 29,00 | 2,00 |
|  | 18,0 | 2 | 15,00 | 8,00 | 11,00 | 2,00 |
|  | 18,0 | 2 | 24,00 | 14,00 | 5,00 | 1,00 |
|  | 18,0 | 2 | 19,00 | 25,00 | 29,00 | 1,00 |
|  | 18,0 | 2 | 14,00 | 13,00 | 30,00 | 1,00 |
|  | 18,0 | 2 | 26,00 | 12,00 | 24,00 | 2,00 |
|  | 18,0 | 2 | 19,00 | 26,00 | 27,00 | 1,00 |
|  | 17,0 | 2 | 21,00 | 22,00 | 24,00 | 1,00 |
|  | 17,0 | 2 | 19,00 | 11,00 | 19,00 | 1,00 |
|  | 17,0 | 2 | 25,00 | 18,00 | 26,00 | 2,00 |
|  | 18,0 | 1 | 8,00 | 14,00 | 13,00 | 1,00 |
|  | 18,0 | 2 | 22,00 | 11,00 | 16,00 | 1,00 |
|  | 17,0 | 1 | 24,00 | 29,00 | 28,00 | 1,00 |
|  | 17,0 | 2 | 21,00 | 6,00 | 15,00 | 1,00 |
|  | 17,0 | 2 | 27,00 | 22,00 | 25,00 | 3,00 |
|  | 17,0 | 1 | 27,00 | 21,00 | 19,00 | 1,00 |
|  | 17,0 | 2 | 27,00 | 11,00 | 15,00 | 1,00 |
|  | 17,0 | 2 | 17,00 | 6,00 | 23,00 | 1,00 |
|  | 17,0 | 2 | 11,00 | 13,00 | 18,00 | 1,00 |
|  | 17,0 | 2 | 17,00 | 12,00 | 25,00 | 2,00 |
|  | 17,0 | 2 | 9,00 | 6,00 | 23,00 | 1,00 |
|  | 18,0 | 1 | 15,00 | 18,00 | 23,00 | 1,00 |
|  | 18,0 | 2 | 10,00 | 10,00 | 14,00 | 1,00 |
|  | 17,0 | 2 | 9,00 | 14,00 | 9,00 | 3,00 |
|  | 17,0 | 1 | 21,00 | 10,00 | 31,00 | 1,00 |
|  | 18,0 | 2 | 24,00 | 19,00 | 28,00 | 1,00 |
|  | 18,0 | 1 | 14,00 | 8,00 | 21,00 | 1,00 |
|  | 18,0 | 1 | 23,00 | 8,00 | 26,00 | 1,00 |
|  | 17,0 | 2 | 14,00 | 27,00 | 31,00 | 1,00 |
|  | 17,0 | 2 | 14,00 | 15,00 | 27,00 | 1,00 |
|  | 18,0 | 2 | 20,00 | 15,00 | 16,00 | 2,00 |
|  | 17,0 | 2 | 32,00 | 32,00 | 32,00 | 1,00 |
|  | 18,0 | 1 | 8,00 | 18,00 | 20,00 | 1,00 |
|  | 18,0 | 2 | 14,00 | 18,00 | 18,00 | 1,00 |
|  | 17,0 | 2 | 28,00 | 28,00 | 18,00 | 2,00 |
|  | 17,0 | 2 | 24,00 | 19,00 | 23,00 | 3,00 |
|  | 18,0 | 2 | 29,00 | 12,00 | 18,00 | 1,00 |
|  | 17,0 | 2 | 10,00 | 19,00 | 22,00 | 1,00 |
|  | 17,0 | 2 | 32,00 | 26,00 | 10,00 | 2,00 |
|  | 17,0 | 2 | 16,00 | 12,00 | 23,00 | 2,00 |
|  | 17,0 | 2 | 15,00 | 25,00 | 30,00 | 2,00 |
|  | 17,0 | 2 | 13,00 | 12,00 | 13,00 | 2,00 |
|  | 17,0 | 2 | 26,00 | 7,00 | 8,00 | 2,00 |
|  | 17,0 | 2 | 12,00 | 20,00 | 26,00 | 1,00 |
|  | 17,0 | 2 | 19,00 | 28,00 | 30,00 | 3,00 |
|  | 18,0 | 2 | 9,00 | 4,00 | 6,00 | 1,00 |
|  | 17,0 | 2 | 11,00 | 8,00 | 19,00 | 2,00 |
|  | 17,0 | 2 | 22,00 | 18,00 | 19,00 | 1,00 |
|  | 17,0 | 1 | 5,00 | 8,00 | 15,00 | 1,00 |
|  | 17,0 | 2 | 28,00 | 22,00 | 24,00 | 1,00 |
|  | 17,0 | 1 | 29,00 | 28,00 | 24,00 | 1,00 |
|  | 18,0 | 1 | 4,00 | 25,00 | 31,00 | 1,00 |
|  | 18,0 | 1 | 20,00 | 9,00 | 11,00 | 1,00 |
|  | 17,0 | 2 | 16,00 | 19,00 | 22,00 | 1,00 |
|  | 18,0 | 2 | 28,00 | 17,00 | 20,00 | 1,00 |
|  | 17,0 | 1 | 9,00 | 15,00 | 16,00 | 1,00 |
|  | 17,0 | 2 | 20,00 | 10,00 | 13,00 | 1,00 |
|  | 17,0 | 1 | 25,00 | 20,00 | 30,00 | 1,00 |
|  | 17,0 | 2 | 12,00 | 12,00 | 18,00 | 2,00 |
|  | 17,0 | 2 | 23,00 | 13,00 | 16,00 | 1,00 |
|  | 17,0 | 2 | 9,00 | 22,00 | 21,00 | 2,00 |
|  | 17,0 | 2 | 21,00 | 14,00 | 21,00 | 2,00 |
|  | 17,0 | 2 | 14,00 | 11,00 | 24,00 | 1,00 |
|  | 17,0 | 2 | 26,00 | 22,00 | 21,00 | 3,00 |
|  | 18,0 | 2 | 13,00 | 11,00 | 10,00 | 3,00 |
|  | 17,0 | 2 | 25,00 | 12,00 | 11,00 | 1,00 |
|  | 17,0 | 2 | 19,00 | 13,00 | 15,00 | 2,00 |
|  | 17,0 | 1 | 14,00 | 21,00 | 27,00 | 2,00 |
|  | 17,0 | 2 | 11,00 | 9,00 | 19,00 | 1,00 |
|  | 17,0 | 2 | 27,00 | 25,00 | 23,00 | 1,00 |
|  | 18,0 | 2 | 19,00 | 18,00 | 24,00 | 1,00 |
|  | 17,0 | 2 | 23,00 | 21,00 | 23,00 | 1,00 |
|  | 17,0 | 2 | 18,00 | 11,00 | 12,00 | 1,00 |
|  | 12,0 | 2 | 20,00 | 5,00 | 14,00 | 1,00 |
|  | 12,0 | 1 | 20,00 | 24,00 | 30,00 | 1,00 |
|  | 12,0 | 1 | 26,00 | 29,00 | 25,00 | 1,00 |
|  | 13,0 | 1 | 13,00 | 21,00 | 29,00 | 1,00 |
|  | 12,0 | 1 | 12,00 | 15,00 | 16,00 | 1,00 |
|  | 12,0 | 1 | 13,00 | 19,00 | 22,00 | 1,00 |
|  | 12,0 | 2 | 23,00 | 13,00 | 23,00 | 1,00 |
|  | 12,0 | 1 | 26,00 | 23,00 | 32,00 | 1,00 |
|  | 12,0 | 1 | 11,00 | 22,00 | 32,00 | 1,00 |
|  | 12,0 | 1 | 20,00 | 14,00 | 13,00 | 1,00 |
|  | 12,0 | 1 | 16,00 | 30,00 | 26,00 | 1,00 |
|  | 12,0 | 1 | 4,00 | 6,00 | 8,00 | 1,00 |
|  | 12,0 | 2 | 26,00 | 19,00 | 30,00 | 1,00 |
|  | 12,0 | 1 | 28,00 | 28,00 | 28,00 | 1,00 |
|  | 12,0 | 1 | 8,00 | 14,00 | 16,00 | 1,00 |
|  | 12,0 | 1 | 17,00 | 24,00 | 24,00 | 1,00 |
|  | 12,0 | 1 | 11,00 | 17,00 | 18,00 | 1,00 |
|  | 12,0 | 1 | 21,00 | 19,00 | 22,00 | 1,00 |
|  | 13,0 | 1 | 15,00 | 13,00 | 20,00 | 1,00 |
|  | 12,0 | 2 | 16,00 | 16,00 | 9,00 | 1,00 |
|  | 12,0 | 1 | 16,00 | 16,00 | 20,00 | 1,00 |
|  | 12,0 | 1 | 20,00 | 23,00 | 18,00 | 1,00 |
|  | 12,0 | 1 | 16,00 | 22,00 | 24,00 | 1,00 |
